# The role of gut microbiota and its metabolites in preventing oncogenesis

**DOI:** 10.3389/fcell.2026.1790063

**Published:** 2026-02-24

**Authors:** Zehua Li, Jiaxi Yan, Ziyi Zeng, Linyong Zhao

**Affiliations:** 1 Department of Plastic and Burn Surgery, West China Hospital, Sichuan University, Chengdu, China; 2 West China School of Medicine, Sichuan University, Chengdu, China; 3 Department of Neonatology, West China Second University Hospital of Sichuan University, Chengdu, China; 4 Department of General Surgery and Gastric Cancer Center, West China Hospital, Sichuan University, Chengdu, China

**Keywords:** cancer prevention, carcinogen detoxification, fecal microbiota transplantation, gut microbiota, immune modulation, oncogenesis, probiotics

## Abstract

The gut microbiota is increasingly recognized as a key determinant of cancer susceptibility, functioning as a dynamic interface between environmental exposures and host physiology. Dysbiosis disrupts immune homeostasis, epithelial integrity, and metabolic equilibrium, thereby fostering a microenvironment conducive to oncogenesis. Conversely, a balanced microbial ecosystem and its metabolites exert potent anti-tumor effects through immune modulation, maintenance of mucosal barrier function, and detoxification of carcinogens. This Review synthesizes emerging mechanistic insights into how commensal microbes and their metabolic products coordinate host defense pathways to suppress malignant transformation. We further discuss translational strategies—ranging from probiotics, prebiotics, and synbiotics to fecal microbiota transplantation and dietary interventions—that leverage microbiome modulation for cancer prevention. Despite compelling preclinical evidence, clinical translation remains constrained by inter-individual variability and incomplete mechanistic understanding. Integration of multi-omics analyses, gnotobiotic models, and precision microbial engineering offers a path toward microbiota-based interventions as a cornerstone of personalized cancer prevention and immunomodulation.

## 1 Introduction

The human gut microbiota refers to the diverse community of microorganisms, including bacteria, archaea, viruses, and fungi, that reside in the gastrointestinal tract (Sender et al., 2016). This highly diverse microbial community has co-evolved with the human host over millennia, confers a broad spectrum of physiological benefits, including nutrient metabolism, immune modulation, and the maintenance of mucosal integrity (Backhed et al., 2005; Wu and Wu, 2012; Hill and Artis, 2010). Perturbations of this equilibrium, termed dysbiosis, have been increasingly implicated in the pathogenesis of a wide range of chronic and multifactorial diseases (Ivleva and Grivennikov, 2022). Over the past two decades, research on the gut microbiome has expanded substantially, driven by advances in next-generation sequencing, computational pipelines tailored to microbiome data, and experimental platforms enabling hypothesis testing at both high- and low-throughput (Bharti and Grimm, 2021; Wensel et al., 2022; Li et al., 2024). In parallel, and as a direct consequence of these developments, microbes have been increasingly recognized as pivotal mediators linking immune responses with cancer development.

Oncogenesis, the process underlying cancer development, results from the complex interplay of genetic predispositions, environmental exposures, and lifestyle factors that collectively promote uncontrolled cellular proliferation and tumor progression (Hanahan and Weinberg, 2011). Although accumulating evidence suggests that dysbiosis of the gut microbiota and its metabolites are associated with oncogenesis, significant limitations remain in its interpretation (Zhao et al., 2023). Notably, dysbiosis does not represent an absolute or universally defined state; instead, its characteristics are highly dependent on both the host and the specific disease context (Scott et al., 2019). Therefore, when analyzing the landscape of the gut and intratumoral microbiome and its metabolites, it is essential to account for inter-patient variability to elucidate the mechanisms underlying cancer initiation (Wong and Yu, 2023; El and Garrett, 2023). At the molecular level, these mechanisms are multifaceted and can be broadly categorized into genomic integration, genotoxicity, chronic inflammation, immune dysregulation and metabolic reprogramming.

The rationale for examining gut microbiota and its metabolites as a promising strategy for cancer prevention lies in its dual role as a regulator of host physiology and disease susceptibility (Gou et al., 2024; Yu et al., 2024). By maintaining immune surveillance, certain microbial environments may reduce cancer risk and prevent oncogenesis.

In this review, we examine recent studies investigating the protective roles of the gut microbiota in carcinogenesis. We highlight synergistic anti-oncogenic mechanisms driven by microbes, with particular emphasis on microbially mediated immune modulation, maintenance of epithelial barrier integrity, and detoxification of carcinogens. Specifically, we address how diverse immune cell populations interact with host microbial communities to prevent chronic inflammation, how commensal microbes preserve mucosal immune homeostasis, and how microbial metabolites contribute to cancer prevention. Furthermore, we evaluate both the potential and the limitations of microbiota- and metabolite-based interventions for personalized cancer suppression, including probiotics, prebiotics, fecal microbiota transplantation, and dietary modulation. Finally, we underscore the many unresolved aspects of microbiota research in oncogenesis and outline potential directions for advancing this rapidly evolving field.

## 2 Microbiota dysbiosis and cancer risk

Dysbiosis, broadly defined as an abnormal alteration in the composition or function of the gut microbiota and its metabolites, has been increasingly linked to elevated cancer risk. Rather than representing a uniform condition, dysbiosis can disrupt host–microbe interactions in ways that vary across individuals and disease contexts. Emerging evidence demonstrates that such disruptions can profoundly alter metabolic activity, immune regulation, and epithelial barrier integrity, thereby fostering a microenvironment conducive to oncogenesis (Ivleva and Grivennikov, 2022; He et al., 2025; Nobels et al., 2025; Schwabe and Jobin, 2013; Francescone et al., 2014). Here, we selected colorectal cancer (CRC), hepatocellular carcinoma (HCC), gastric cancer (GC), and breast cancer (BC) as representative malignancies because they illustrate direct, axis-mediated, pathogen-associated, and endocrine-driven mechanisms of microbiota-associated carcinogenesis.

### 2.1 Colorectal cancer (CRC)

Multiple studies have reported that the development of CRC is closely linked to interactions between various microbes, their metabolites, and the intestinal epithelial barrier (Herlo et al., 2024; Wu et al., 2024). Specifically, the dysbiosis can lead to a pro-oncogenic environment, resulting in chronic inflammation and immune dysregulation (Wong and Yu, 2023). Consequently, clinical cohort studies of CRC patients exhibit enrichment of pro-oncogenic taxa such as *Fusobacterium nucleatum*, enterotoxigenic *Bacteroides fragilis*, and *pks* ^*+*^ *Escherichia coli* (Wang and Fang, 2023; Sears and Pardoll, 2011; Jans and Vereecke, 2025) and depletion of beneficial gut bacteria such as *Bifidobacterium*, *Clostridium butyricum*, *Faecalibacterium prausnitzii*, *Roseburia intestinalis*, and *Streptococcus thermophilus* (Lopez-Siles et al., 2017; Kang et al., 2023; Correa et al., 2005). Preclinical fecal microbiota transplantation (FMT) studies have further validated the causal role of gut microbiome dysbiosis in CRC development. Germ-free (GF) mice receiving microbiota from CRC patients exhibited pronounced tumor-promoting effects, such as enhanced epithelial cell proliferation, increased polyp formation, greater dysplasia, and elevated inflammatory markers, compared with those receiving transplants from healthy donors (Wong et al., 2017). Nevertheless, the causal relationship between specific gut bacterial species and the emergence of oncogenic somatic mutations remains to be fully elucidated, although an increasing number of mechanistic studies have provided compelling evidence supporting this association.

### 2.2 Hepatocellular carcinoma (HCC)

Evidence from both clinical studies and preclinical animal models indicates that dysbiosis influences hepatocarcinogenesis through bidirectional communication along the gut–liver axis. In a preclinical study investigating fecal biomarkers, significant alterations in gut microbiota composition were observed during the progression of liver disease, characterized by markedly increased abundances of *Bacteroides* spp., *Clostridium cocleatum*, and *Desulfovibrio* spp., which correlated with elevated circulating LPS levels (Xie et al., 2016). LPS-mediated activation of TLR4 signaling in hepatocytes and Kupffer cells was shown to promote chronic hepatic injury and tumorigenesis (Jing et al., 2012). Collectively, these findings underscore the pivotal role of the gut microbiota in the progression and pathogenesis of hepatocellular carcinoma.

Bacterial metabolites and endotoxins circulating in the portal vein serve as critical mediators of hepatic inflammation, fibrosis and subsequent HCC progression, as their levels correlate with the severity of intestinal barrier disruption and microbial dysbiosis (Albillos et al., 2020; Guan et al., 2022). A compelling example is that the deficiency of the inflammasome sensor NLRP6 in mice leads to a dysbiotic gut microbiota and hepatic inflammatory microenvironment. This dysbiosis promotes hepatocellular carcinoma development by shaping the hepatic inflammatory microenvironment through activation of TLR4 and TLR9 signaling (Schneider et al., 2022). Another study reported a reduced abundance of *Bacteroides thetaiotaomicron* in patients with recurrent hepatocellular carcinoma (HCC). Further analysis revealed that acetic acid produced by *B. thetaiotaomicron* can modulate macrophage polarization toward the M1 phenotype, thereby enhancing the cytotoxic activity of CD8^+^ T cells and inhibiting HCC tumor growth (Ma et al., 2024).

### 2.3 Gastric cancer (GC)

It has been widely reported that *Helicobacter pylori* (*H.pylori*) infection plays a crucial role in the initial steps of carcinogenesis by exacerbate chronic inflammation and progressive degradation of the architecture and function of the gastric epithelium (Chen et al., 2013).

In general, gastric cancer–associated dysbiosis is characterized by a microbial community with genotoxic potential, marked by reduced microbial diversity, decreased *Helicobacter* abundance, and enrichment of newly emerging or opportunistic bacterial genera (Coker et al., 2018; Zeng et al., 2024; Lei et al., 2024). While *H. pylori* remains the most established microbial risk factor, non-*H. pylori* microbiota also play significant roles in gastric carcinogenesis (Liu et al., 2022). By promoting M2 macrophage polarization through activation of the TLR4/PI3K/Akt signaling pathway, *Propionibacterium acnes* may act as a potential contributor to gastric cancer progression in addition to *H. pylori*. A recent study further suggested that *F. nucleatum* infection increases the secretion of exosomes by gastric cancer (GC) cells, thereby promoting GC progression (Li et al., 2021a). Notably, although overall α-diversity decreases in carcinoma tissues, increased abundances of non-*Helicobacter Proteobacteria*, *Firmicutes*, and *Actinobacteria* have been detected in gastric cancer specimens (Ferreira et al., 2018). Moreover, the gastric cancer–associated microbiota exhibits enhanced nitrate and nitrite reductase activities, facilitating the formation of N-nitroso compounds—potent mutagens that accelerate gastric carcinogenesis.

In summary, while *H*. *pylori* remains the principal bacterial agent associated with gastric cancer, broader dysbiosis within the gastrointestinal microbiota also play a substantial role in GC progression.

### 2.4 Breast cancer (BC)

Emerging evidence indicates that the microbiota, both within the gut and the mammary gland, exerts significant influence over BC pathogenesis through metabolic, endocrine, and immunological pathways (Plaza-Diaz et al., 2019). Of these associations, the link between breast cancer and the microbial “estrobolome” has received the greatest research attention (Sui et al., 2021). It is a consortium of microbial genes responsible for estrogen metabolism (Ervin et al., 2019). Clinical observational studies and mechanistic *in vitro* analyses suggest that microbial imbalance, via heightened β-glucuronidase activity, enhances the reactivation of conjugated estrogens, thereby increasing systemic estrogen exposure and promoting estrogen receptor–positive tumor development (Arnone and Cook, 2022). Studies examining the relationship between gut microbial diversity and estrogen levels in postmenopausal women with BC have shown that variability in gut microbiota composition is associated with differences in estrogen metabolism and circulating estrogen concentrations. Specifically, clinical studies in postmenopausal women have shown that lower microbial diversity correlates with elevated systemic estrogen levels, suggesting that reduced gut microbial diversity may contribute to an increased risk of breast cancer (Wu et al., 2023; Flores et al., 2012).

Microbiota dysbiosis has emerged as a unifying yet context-specific factor in cancer development. Across multiple malignancies, disruptions in microbial composition and metabolism promote chronic inflammation, epithelial barrier dysfunction, and genotoxic stress. Collectively, these findings position gut microbiota imbalance as a central modulator of oncogenesis and a promising therapeutic target. Conversely, when in equilibrium, the gut microbiota plays an equally vital role in maintaining host homeostasis and preventing tumor initiation. Thus, the relationship between the microbiota and cancer is inherently dualistic: while the loss of microbial diversity and function can drive oncogenesis, a stable and metabolically active microbiome can confer protection.

## 3 Mechanisms by which gut microbiota and its metabolites prevent oncogenesis

### 3.1 Rationale for microbial focus in oncogenesis

The gut microbiota acts as a critical interface connecting environmental carcinogens and host susceptibility to cancer (Azcarate-Peril et al., 2011). Metagenomic studies show cancer patients have reduced gut microbiota diversity, especially fewer butyrate-producing bacteria, versus healthy individuals. The dysbiosis, characterized by diminished community stability, heightens exposure to carcinogens and promotes maladaptive immune responses (Bhatt et al., 2017; Garrett, 2015). Importantly, microbial metabolites play a dual role in both promoting and suppressing cancer development, positioning gut microbiota regulation as a promising strategy for cancer prevention (Bose and Mukherjee, 2019). The profound influence of gut microbiota on host metabolism highlights their potential as modifiable targets for interrupting carcinogenic pathways (Hanus et al., 2021). Specifically, the gut microbiota prevent oncogenesis through synergistic mechanisms, including modulation of the immune system, preservation of epithelial barrier integrity, and detoxification of carcinogens. Together, these processes reduce the risk of DNA damage and chronic inflammation—key drivers of tumorigenesis (Tsilimigras et al., 2017; Sun et al., 2025).

### 3.2 Immune system modulation

The gut microbiota regulates systemic immunity through interacting with host cells and influencing both innate and adaptive immune responses essential for protecting against precancerous lesions (Garrett, 2015). Microbial metabolites, such as short-chain fatty acids (SCFAs) and polyamines, function as immune signaling molecules that regulate inflammatory pathways, contributing to prevent chronic inflammation—a known precursor to DNA damage and cancer development (Tsilimigras et al., 2017; Yu and Zhong, 2022). Dysbiosis disrupts this immune balance, evident in the skewed Th17/Treg ratio and diminished cytotoxic activity, both are directly associated with increased cancer risk across multiple organs. Maintaining microbial balance is therefore critical for sustaining immune function and reducing cancer risk (Levy et al., 2017). This balanced immunity not only eliminates oncogenic agents but also restrains excessive inflammation, thereby blocking carcinogenic pathways.

#### 3.2.1 Foundational immune programming

Studies in GF mouse models demonstrate that the absence of microbiota leads to underdeveloped mucosal immunity, characterized by fewer Peyer’s patches, IgA^+^ plasma cells, and CD8^+^ intraepithelial lymphocytes, weakening their ability to detect and eliminate oncogenic viruses and bacteria; these defects can be reversed by colonizing the microbiota (Chung and Kasper, 2010).

Pattern recognition receptors (PRRs) sense microbe-associated molecular patterns (MAMPs) from commensals, shaping host immune responses (Chu and Mazmanian, 2013). Early exposure to microbiota adjusts mononuclear phagocytes through chromatin remodeling, enhancing systemic antiviral and antibacterial immunity to eliminate potential tumor cells (Ganal et al., 2012; Takiishi et al., 2017). Type I interferons (IFN-I) and NK cells are central to antiviral immunity: IFN-I activates DCs to promote NK and CD8^+^ T cell activity, while NK cells eliminate MHC-I-deficient tumor cells via perforin/granzyme or Fas/FasL pathways (Kawai and Akira, 2006; Tougeron et al., 2013). Commensal microbiotas prime these responses—critical since ∼10% of cancers (e.g., HPV-induced cervical cancer, HBV-induced HCC) stem from viral infections, and robust antiviral immunity clears infected cells before malignant transformation (Schiller and Lowy, 2021; Plummer et al., 2016; Winkler and Thackray, 2019).

This early immune programming lays the foundation for long-term immune competence, helping to prevent chronic inflammation that fosters the formation of a tumor-promoting microenvironment.

#### 3.2.2 Anti-tumor immunity enhancement

##### 3.2.2.1 Treg cells

Regulatory T cells (Tregs) suppress excessive inflammation and maintain immune tolerance, thereby inhibiting chronic inflammation-driven tumorigenesis (Levy et al., 2017; Arpaia et al., 2013). Studies show that *Bifidobacterium infantis* expands CD4^+^CD25^+^Foxp3^+^ Tregs to suppress inflammation-related cancer (O'Mahony et al., 2008). Indigenous *Clostridium* species (clusters IV/XIVa) expand colonic Tregs by inducing TGF-β activation and promoting IL-10^+^CTLA4^high^ iTreg differentiation, which protects against colitis and systemic IgE responses (Atarashi et al., 2011). Microbial-derived SCFAs (acetate/propionate/butyrate) further enhance Treg numbers, function, and Foxp3/IL-10 expression via GPR43 (Ffar2)-dependent HDAC inhibition, restoring colonic Treg homeostasis and ameliorating inflammation in mice (Smith et al., 2013). Conversly, Dysbiosis disrupts this Treg-Th17 balance, enabling inflammation and tumor progression (Zhang et al., 2019).

##### 3.2.2.2 γδ T cells

γδ T cells contribute to tumor prevention through direct cytotoxicity (granzyme/perforin) and IFN-γ-mediated antitumor activity (Hannani et al., 2012; Kong et al., 2009). However, IL-17-producing γδ T cells (γδT17) promote tumor progression by driving immunosuppressive PMN-MDSC and TAN accumulation via IL-17/GM-CSF secretion (Wu et al., 2014; Coffelt et al., 2015). Commensal bacteria maintain protective γδ intraepithelial lymphocytes (IELs) and critically suppress γδT17 activity via metabolites like SCFAs (Li et al., 2017; Wu et al., 2017; Dupraz et al., 2021). Conversely, microbial dysbiosis impairs barrier function, reduces SCFAs (alleviating γδT17 suppression), and promotes γδT17 polarization, ultimately enabling immunosuppression and tumor development (Jenkins et al., 2019).

##### 3.2.2.3 Th17 cells

*Segmented filamentous bacteria* (SFB) specifically induce Th17 cells, enhancing mucosal defense against pathogens (Ivanov et al., 2009). Commensal microbiota collectively modulate Th17 cell differentiation via TGF-β/IL-6 signaling (Chung and Kasper, 2010; Zhang et al., 2019).

##### 3.2.2.4 Th22 cells

SFB induce Th22 cell differentiation independent of classical Th17 cells and TGF-β signaling. Experimental results demonstrate that SFB colonization significantly enhances IL-22^+^CD4^+^ T cell numbers during infection, confirming that specific gut microbiota modulate Th22 development to strengthen mucosal defense. Th22 cells may reduce cancer risk by secreting IL-22 to enhance intestinal barrier integrity and suppress chronic inflammation (Roy et al., 2021).

##### 3.2.2.5 Th1/Th2 balance

*Bacteroides fragilis* monocolonization corrects Th1/Th2 imbalances in GF mice. By maintaining T-cell homeostasis and suppressing pathological inflammation, these microbiota-mediated immunomodulatory effects collectively reduce risks of inflammation-driven carcinogenesis (Zhang et al., 2019).

##### 3.2.2.6 Natural killer (NK) cells

Natural killer (NK) cells directly recognize and eliminate tumor cells, playing a critical role in antitumor immunity. *In vitro* studies have shown that gut microbiota-derived short-chain fatty acids (SCFAs) enhance NK cell cytotoxicity against tumors like multiple myeloma by reducing anti-inflammatory IL-10 secretion and promoting extracellular vesicle release (Perez et al., 2024). Conversely, intestinal microbiota suppresses intratumoral NK cell infiltration and IFNγ expression in pancreatic cancer, accelerating tumor progression (Yu et al., 2022).

##### 3.2.2.7 Natural killer T (NKT) cells

**FIGURE 1:**
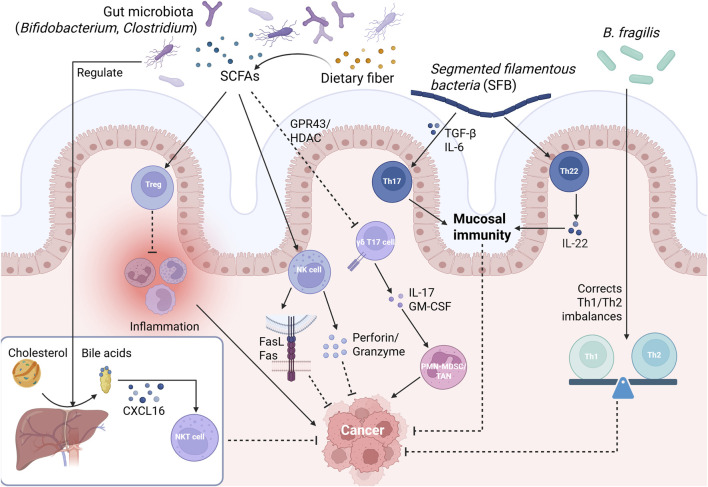
The Interplay Between the Gut Microbiome, Its Metabolites, and Immune Cells. Commensal microbiota (*Bifidobacterium, Clostridium*) produce short-chain fatty acids (SCFAs) that exert coordinated immunoregulatory effects by: Sender et al. (2016) expanding Treg cells via GPR43-mediated HDAC inhibition to suppress inflammation; Backhed et al. (2005) inhibiting the pro-tumor γδT17-PMN-MDSC/TAN axis; Wu and Wu (2012) enhancing NK cell cytotoxicity. Concurrently, *segmented filamentous bacteria (SFB)* induce Th17 cell differentiation via TGF-β/IL-6 signaling and drive Th22 cell-mediated IL-22 secretion, thereby reinforcing mucosal immunity. Separately, microbiome-processed primary bile acids (e.g., CDCA) activate the CXCL16-CXCR6 axis to recruit hepatic NKT cells for tumor surveillance. Additionally, *Bacteroides fragilis* monocolonization corrects Th1/Th2 imbalances in germ-free hosts to reduce carcinogenesis risk. Collectively, these pathways orchestrate anti-tumor immunity by preventing chronic inflammation, preserving epithelial barrier integrity, and enabling direct elimination of transformed cells. Notes: Solid arrows (→) indicate activation or induction of the immediate downstream process. Blunted lines (---) indicate inhibition or suppression of the immediate downstream target.

NKT cells are essential for liver antitumor surveillance against primary and metastatic tumors. Gut microbiome-regulated bile acid metabolism controls hepatic NKT cell accumulation via the CXCL16-CXCR6 axis: primary bile acids (e.g., CDCA) induce CXCL16 on liver sinusoidal endothelial cells, promoting NKT cell recruitment and tumor suppression, while secondary bile acids (e.g., ω-MCA) inhibit this pathway (Ma et al., 2018) (Figure 1).

### 3.3 Maintenance of epithelial barrier integrity and mucosal immunity

The intestinal epithelial barrier, consisting of physical, chemical, and immunological components, prevents harmful substances, such as carcinogens and pathogenic bacteria, from entering the body (Bose and Mukherjee, 2019). Disruption of this barrier facilitates bacterial translocation and triggers systemic inflammation, which can induce DNA damage and promote cancer development (Schwabe and Jobin, 2013; Yu and Zhong, 2022). The gut microbiota and its metabolites help maintain the integrity of the epithelial barrier and mucosal immunity, thereby lowering cancer risk. Dysbiosis-induced barrier dysfunction, which increases carcinogen exposure and amplifies inflammatory responses, underscores the importance of maintaining microbial homeostasis for cancer prevention.

#### 3.3.1 Intestinal barrier formation and maintenance

The barrier regulates luminal factor-immune interactions; disruptions of this barrier lead to “leaky gut,” which has been linked to IBD, metabolic diseases, and colorectal cancer (Neurath et al., 2025).

Commensals fortify the physical barrier: The physical barrier is formed by intestinal epithelial cells with tight junctions, controlling permeability and preventing microbial translocation. *Bacteroides thetaiotaomicron* upregulates tight junction proteins (claudin, occludin), while butyrate activates AMPK to repair tight junctions (Takiishi et al., 2017; Miao et al., 2016; Groschwitz and Hogan, 2009). Besides, Impaired intestinal barrier function permits microbiota access to epithelial TLRs, activating calcineurin-NFATc3 signaling. This pathway promotes cancer stem cell survival and proliferation, driving tumor development. Inhibiting microbiota-TLR interactions or blocking calcineurin-NFATc3 activation suppresses tumorigenesis, highlighting barrier integrity as a critical preventive mechanism (Peuker et al., 2016).

Chemical barrier maintenance: The chemical barrier comprises mucins, antimicrobial peptides, and secretory IgA, which inhibit microbes through secretion and immune exclusion. Microbialstimuli (e.g., SFB) induce mucin (MUC2) and AMP production; *Lactobacillus* and *Bifidobacterium* increase mucus viscosity to block carcinogens (Bhatt et al., 2017; Johansson et al., 2014). Gut microbiota-produced polyamines (e.g., putrescine, spermidine) enhance intestinal barrier integrity and suppress procarcinogenic inflammation, thereby inhibiting tumor initiation (Murray Stewart et al., 2018; Ramos-Molina et al., 2019).

#### 3.3.2 Mucosal immunity

The maintenance of spatial homeostasis within the gut mucosal immune barrier prevents bacterial penetration and aberrant inflammatory responses. Key cellular and molecular players contribute to this homeostasis:

Cellular Effectors: Dendritic cells in the lamina propria sample luminal antigens to coordinate IgA secretion, thereby restricting bacterial translocation and maintaining barrier integrity (Gorski and Weber-Dabrowska, 2005). Concurrently, butyrate promotes the production of IL-22 by group 3 innate lymphoid cells (ILC3s), enhancing mucosal defense and epithelial repair.

Cytokine Regulation and Microbiota Control: Study indicates that IL-33 gene deficiency is associated with an increased risk of cancer; this susceptibility arises from the resultant dysbiotic microbiota (characterized by expansion of mucolytic bacteria like *Akkermansia muciniphila*), which promotes epithelial damage, early release of proinflammatory IL-1α, and ultimately drives colitis and tumorigenesis (Malik et al., 2016). Lipocalin 2 (LCN2), induced by the microbiota via MYD88, further reinforces this barrier by limiting bacterial iron acquisition and preventing the formation of colitogenic microbiota, thus reducing colorectal cancer risk (Singh et al., 2016; Moschen et al., 2016).

Avoiding Barrier Dysregulation: Conversely, excessive TLR4 expression, as seen in inflammatory bowel disease (IBD) or colitis-associated cancer (CAC), disrupts barrier function and fosters a proinflammatory microbiota, exacerbating colitis and tumorigenesis (Yu and Zhong, 2022; Dheer et al., 2016).

Disruption of this immune barrier homeostasis has dire consequences. When the spatial integrity is compromised (e.g., due to pathogens like *H. pylori*), bacterial penetration occurs. This triggers the release of potent proinflammatory cytokines (e.g., IL-6, TNF-α) and causes chronic mucosal damage. This persistent inflammation is a key oncogenic driver: it directly mediates cellular damage and proliferation conducive to cancer, and establishes a microenvironment of chronic damage (e.g., *H. pylori-*induced gastritis, a recognized precancerous lesion), significantly increasing the risk of malignancies like gastric adenocarcinoma (Fox and Wang, 2007).

**FIGURE 2:**
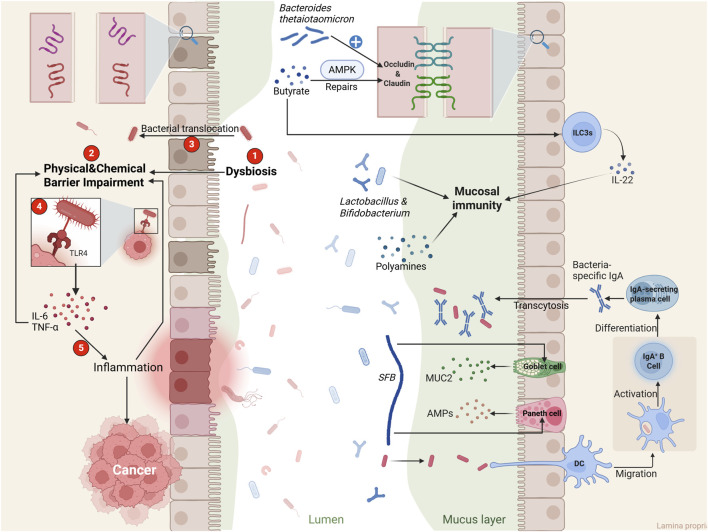
The Distinct Roles of Gut Microbial Homeostasis and Dysbiosis in Regulating Epithelial Barrier Integrity and Mucosal Immunity. This schematic contrasts the consequences of a balanced versus dysregulated gut microbiota. Beneficial commensal bacteria are depicted in blue, and potentially harmful bacteria in red. The right panel (“Normal condition”) illustrates a healthy microbiota-dominated state that maintains homeostasis. The left panel (“Dysbiotic condition”) depicts microbial imbalance characterized by the overgrowth of harmful bacteria, which initiates a pathological cascade. Under physiological conditions, a balanced microbiota and its metabolites (e.g., butyrate, polyamines) fortify the epithelial barrier by upregulating tight junction proteins, activating AMPK activation, and enhancing mucus production. Dendritic cell (DC)-coordinated secretion of secretory IgA (sIgA) limits bacterial translocation, while butyrate-induced IL-22 production from group 3 innate lymphoid cells (ILC3s) promotes mucosal defense and repair. Conversely, dysbiosis initiates a vicious cycle. It impairs barrier function through tight junctions disassembly and mucolysis, allowing bacterial translocation. This leads to TLR4 hyperactivation, chronic inflammation, and progressive barrier damage, collectively driving tumorigenesis.

Therefore, the gut microbiota’s role in actively preserving the spatial homeostasis of the immune barrier is fundamental to cancer prevention. By preventing bacterial encroachment and the ensuing cascade of abnormal inflammation and chronic tissue damage, this equilibrium significantly reduces cancer risk (Figure 2).

### 3.4 Detoxification and metabolism of carcinogens

The gut microbiota participates in the metabolism and detoxification of carcinogens, thereby influencing cancer development (Sun et al., 2025; Louis et al., 2014). By producing specific enzymes, gut bacteria can transform carcinogens into less harmful substances or promote their excretion, reducing the damage to host cells. Conversely, dysbiosis may impair these detoxification processes, enhauncing carcinogen activity and increasing cancer risk. Therefore, maintaining gut microbiota’s normal detoxification and metabolic function is an important way to prevent cancer.

#### 3.4.1 Direct detoxification

Gut microbial metabolism of carcinogens can have dual effects, either mitigating their harmful impact or enhancing their carcinogenicity. Normally, certain bacteria participate in carcinogen degradation. For instance, *Klebsiella* species deaminate melamine; some *E. coli* strains hydrolyze IQ-glucuronide, and gut microbes reduce azo compounds (Koppel et al., 2017; Zheng et al., 2013; Ervin and Redinbo, 2020). Microbial β-glucuronidases can decompose conjugated nitrosamines (such as BBN/EHBN), promoting their excretion in feces and reducing the risk of bladder cancer (Roje et al., 2024). Dysbiosis disrupts normal metabolism, leading to the accumulation of mutagens—such as altered azo reduction that enhances benzidine induced carcinogenesis (Rafii and Cerniglia, 1995).

#### 3.4.2 Metabolite-mediated regulation

SCFAs: Produced by bacterial fermentation of fiber, SCFAs inhibit HDAC to suppress colorectal cancer cell proliferation (Li et al., 2018). They activate GPR43 to secrete IL-18 and AMPs, enhancing barrier function. However, butyrate may promote metastasis in ATP-depleted tumors via β-oxidation (Donohoe et al., 2014; Vipperla and O'Keefe, 2016).

Polyamines: Spermine, spermidine, and putrescine (from amino acid metabolism) support epithelial turnover and lymphocyte activity but can induce DNA damage at high concentrations (Hasko et al., 2000; Pegg, 2013).

Indole-3-lactic acid: A metabolite of *Lactobacillus plantarum* L168, it enhances IL12a production in DCs to prime CD8^+^ T cell immunity against tumors and inhibits Saa3 to improve tumor-infiltrating CD8^+^ T cell function (Zhang et al., 2023).

#### 3.4.3 Bile acid homeostasis

Primary bile acids, synthesized from cholesterol in the liver (e.g., cholic acid, chenodeoxycholic acid), undergo microbial metabolism in the gut—primarily via 7α-dehydroxylase produced by bacteria like *Clostridium scindens*—to form secondary bile acids (e.g., deoxycholic acid [DCA], lithocholic acid [LCA]). This process plays a critical role in regulating host-microbe crosstalk and carcinogenesis (Jia et al., 2018; Collins et al., 2023; Yu et al., 2020). Primary bile acids activate farnesoid X receptor (FXR), maintaining intestinal barrier integrity, inhibiting IL-17-driven inflammation, and regulating bile acid synthesis through negative feedback via fibroblast growth factor 19 (FGF19). FXR knockout mice develop spontaneous liver tumors and show increased colorectal cancer susceptibility, highlighting its tumor-suppressive role (Vavassori et al., 2009; Maran et al., 2009). Secondary bile acids like LCA also activate G-protein-coupled bile acid receptor 1 (TGR5), stimulating anti-inflammatory pathways in macrophages and enhancing energy metabolism to limit carcinogenic microenvironments (Kawamata et al., 2003; Fiorucci and Distrutti, 2015). However, high-fat diets enrich 7α-dehydroxylase-producing bacteria, increasing DCA levels—DCA induces reactive oxygen/nitrogen species (ROS/RNS) to cause DNA damage, provokes senescence-associated secretory phenotype (SASP) in hepatic stellate cells (releasing IL-6 and TNF-α), and promotes obesity-associated hepatocellular carcinoma (HCC) (Payne et al., 2008). Additionally, microbiota-derived bile acids act as androgen receptor (AR) antagonists, promoting stem-like properties of CD8^+^ T cells via CD8^+^ T cell-intrinsic AR signaling to inhibit tumor progression and potentiate anti-PD-1 therapy efficacy (Jin et al., 2025). This bidirectional interplay underscores the critical role of microbial regulation of bile acid metabolism in cancer prevention.

## 4 Therapeutic and preventive potential

**FIGURE 3:**
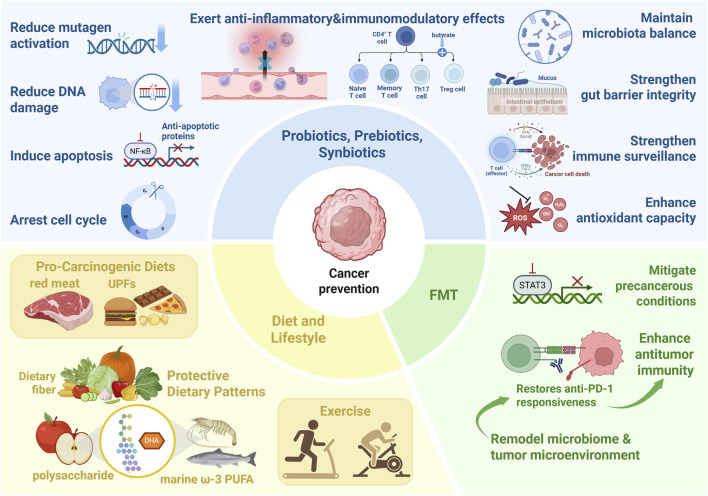
Major Microbiota-Based Approaches to Prevent Oncogenesis. The figure illustrates three major microbiota-targeted intervention approaches. Probiotics, Prebiotics, and Synbiotics exert direct anti-tumor effects (e.g., induction of apoptosis, cell cycle arrest), promote carcinogen detoxification (e.g., reduced DNA damage, inhibition of mutagen activation), modulate immune responses, and strengthen gut barrier integrity. Fecal Microbiota Transplantation (FMT) remodels the host microbiome to mitigate precancerous conditions (e.g., in IBD) and enhances antitumor immunity by restoring responsiveness to immunotherapy. Diet and Lifestyle Interventions highlight the contrasting roles of pro-carcinogenic diets (e.g., red meat, UPFs) versus protective factors (e.g., dietary fiber, omega-3 PUFAs, exercise), acting primarily through modulation of microbial composition and production of protective metabolites like SCFAs. Collectively, these strategies underscore the gut microbiota’s pivotal role as a therapeutic target in cancer prevention and treatment.

The gut microbiota constitutes a pivotal mediator between environmental exposure and host physiology. Emerging evidence indicates that its dysregulation contributes to oncogenesis and tumor development, thereby positioning the gut microbiota as a promising target for therapeutic and preventive cancer interventions. Strategies aimed at modulating microbial composition or regulating metabolite production hold potential to suppress carcinogenesis by enhancing anti-tumor immunity and preserving both intestinal and systemic homeostasis (Gerner and Meyskens, 2004). These insights provide a rationale for the clinical translation of microbiota-targeted approaches, including probiotics and prebiotics, fecal microbiota transplantation, and dietary interventions, as complementary strategies in cancer prevention (Figure 3).

### 4.1 Probiotics, prebiotics and synbiotics

Probiotics are defined as “live microorganisms that, when administered in adequate amounts, confer a health benefit on the host” (Kvakova et al., 2022). A growing body of preclinical evidence supports their potential in cancer prevention through multiple mechanisms, including immunomodulation, carcinogen detoxification, direct antitumor signaling, and microbiota modulation. Prebiotics are “selectively fermented ingredients that allow specific changes in gut microbiota composition/activity”, and their combination with probiotics as synbiotics often yields enhanced efficacy (Bruno-Barcena and Azcarate-Peril, 2015).

#### 4.1.1 Anti-inflammatory and immunomodulatory actions

Probiotics exert notable anti-inflammatory and immunomodulatory effects. Strains such as *Lactobacillus rhamnosus* GG-derived protein p40 activate EGFR signaling, enhance mucin production, and suppress NF-κB-mediated inflammation, thereby interrupting the inflammation-dysplasia-carcinoma sequence (Wang L. et al., 2014). *Lactobacillus gasseri* augments macrophage phagocytosis and promotes S-phase proliferation in RAW264.7 cells via upregulation of PCNA and cyclin A, strengthening immune surveillance (Foo et al., 2011). *Lactobacillus plantarum* ZDY 2013 suppresses Th1/Th17 responses and inhibits pro-inflammatory cytokines in *H. pylori*-infected models, preventing gastric inflammation and microbiota disruption (Pan et al., 2016). In Apc^Min/+^ mice, microencapsulated *Lactobacillus acidophilus* delivered in yogurt increased CD8^+^ T cell infiltration and cleaved caspase-3, indicating enhanced antitumor immunity and apoptosis (Urbanska et al., 2016). Similarly, *Lactobacillus plantarum* stimulated NK and CD8^+^ T cell activity, promoted Th1 response, and upregulated IFN-γ in CT26 murine tumor models (Hu et al., 2015). Certain probiotic mixtures, such as VSL#3, attenuated colitis and tumorigenesis in multiple models by reducing pro-inflammatory cytokines (TNF-α, IL-1β, IL-6) and increasing IL-10 (Talero et al., 2015; Bassaganya-Riera et al., 2012).

Prebiotics such as inulin are fermented by beneficial bacteria like *Bifidobacterium* to produce butyrate, which inhibits histone deacetylases (HDACs) and promotes regulatory T cell (Treg) differentiation, contributing to anti-inflammatory and immunoregulatory effects (Smith et al., 2013; Pool-Zobel et al., 2002).

Synbiotic combinations, including inulin with *L. rhamnosus* and *B. lactis*, further potentiate these benefits by synergistically reducing tumor incidence and suppressing pro-inflammatory mediators, including COX-2 (Femia et al., 2002).

#### 4.1.2 Carcinogen detoxification

Probiotics contribute to chemoprevention by detoxifying dietary carcinogens. *Lactobacillus rhamnosus* Vc directly binds the genotoxic compound MNNG, facilitating its biotransformation into non-mutagenic metabolites (Pithva et al., 2015). *L. rhamnosus* IMC501 reduces PhIP-induced DNA damage in colonocytes, decreases fecal β-glucuronidase and N-acetyl-β-glucosaminidase activities, and modulates microbial enzyme profiles to lower genotoxic metabolite production (Dominici et al., 2014). Probiotic consortia also downregulate procarcinogenic enzymes, including β-glucuronidase and nitroreductase in the colon lumen, thereby reducing mutagen activation (Verma and Shukla, 2013). Moreover, *L. acidophilus, L. casei, and L. lactis* significantly decreased DNA damage and tumor incidence in a DMH-induced rat model, likely through free radical reduction and prevention of DNA adduct formation (Kumar et al., 2010).

Prebiotics such as isomatooligosaccharides (IMOs) reduce fecal β-glucuronidase and β-glucosidase activities, decrease bile acids and amino acids, and inhibit aberrant crypt foci (ACF) formation, demonstrating protective effects against carcinogen-induced tumorigenesis (Chen et al., 2022).

#### 4.1.3 Direct anti-tumor signaling

Several probiotics directly inhibit tumor growth through induction of apoptosis and cell cycle arrest. *Streptococcus thermophilus*-secreted β-galactosidase induces cell cycle arrest in colorectal cancer (CRC) cells via galactose-mediated OXPHOS activation and Hippo pathway suppression (Li et al., 2021b). *Lactococcus lactis* HkyuLL 10 secretes α-mannosidase, which triggers mitochondrial apoptosis via cytochrome C release and ER stress through CHOP upregulation in neoplastic cells (Su et al., 2024). *Lactobacillus salivarius* Ren inhibits AKT phosphorylation and downregulates cyclin D1 and COX-2, thereby inducing apoptosis and cell cycle arrest (Dong et al., 2020). *Lactobacillus paracasei* X12 upregulates pro-apoptotic genes (Bax, Caspase-3, Caspase-9) while downregulates anti-apoptotic genes (Bcl-2, Jak-1, Akt-1), activating both intrinsic and extrinsic apoptotic pathways (Mousavi Jam et al., 2020). *Bacillus polyfermenticus* exhibits strong adherence to intestinal cells and inhibits colon cancer growth in a dose-dependent manner, likely through induction of apoptosis (Lee et al., 2007).

Prebiotics such as oligofructose also reduce colon tumors and enhance gut-associated lymphoid tissue (GALT), suggesting their immunomodulatory role in tumor prevention (Pool-Zobel et al., 2002).

Synbiotics demonstrate enhanced efficacy in inducing apoptosis and suppressing tumorigenesis. For instance, resistant starch combined with *Bifidobacterium lactis* markedly facilitated the acute apoptotic response to genotoxic carcinogens in the distal colon, whereas neither the probiotic nor prebiotic alone was effective (Le Leu et al., 2005). In AOM-induced rats, this synbiotic combination significantly reduced neoplasm incidence and multiplicity, accompanied by increased SCFA production and reduced epithelial cell proliferation, though without altering spontaneous apoptosis (Le Leu et al., 2010). Likewise, formulation such as inulin with *L. rhamnosus* GG and *B. lactis* Bb12, significantly reduced colorectal tumor numbers and malignant progression, while increasing SCFA levels and apoptosis in normal mucosa (Femia et al., 2002).

#### 4.1.4 Antioxidant effects and microbiota modulation

Probiotics also demonstrate antioxidant properties and modulate gut microbial composition. *Lactobacillus paracasei* DTA81 reduces hepatic oxidative stress (MDA, carbonyl protein) and enriches SCFA-producing *Ruminiclostridium* in DMH-induced mice (da Silva Duarte et al., 2020). *Bacillus polyfermenticus* attenuates DMH-induced genotoxicity and oxidative stress while enhancing plasma antioxidant capacity (Park et al., 2007). *L. salivarius* counteracts DMH-induced dysbiosis by increasing *Prevotella* and decreasing *Ruminococcus* and *Bacteroides dorei*, thereby restoring a healthier microbial community (Zhang et al., 2015). *Lactobacillus casei* ATCC 393 reduces aberrant crypt foci (ACF) and downregulates ornithine decarboxylase (ODC), helping regulate polyamine metabolism and exerting antimutagenic effects (Irecta-Najera et al., 2017).

Prebiotics such as dietary fiber and specific plant polysaccharides enrich beneficial bacteria including *Lactobacillus, Bifidobacterium, Roseburia*, and *Eubacterium*, enhancing SCFA production and strengthening gut barrier integrity (So et al., 2018; Guo et al., 2021).

Synbiotic formulations, including *L. acidophilus* with oligofructose/maltodextrin, alter gut microbiota, enhance mucosal barrier function (MUC2, ZO-1, occludin), and decrease pro-carcinogenic signaling pathways (TLR4, COX-2, caspase-3, β-catenin) (Kuugbee et al., 2016).

### 4.2 Fecal microbiota transplantation (FMT)

Fecal microbiota transplantation (FMT) is defined as the transplantation of gut microbiota from healthy donors to recipients via the upper or lower gastrointestinal route, with the aim of restoring microbial diversity and function (Chen et al., 2019). It represents an emerging therapeutic approach for cancer prevention by modulating gut microbial communities to suppress inflammatory, proliferative, and pro-carcinogenic pathways, as well as reducing microbiota-induced genotoxicity.

Currently, FMT is most extensively applied in the treatment of recurrent *Clostridium difficile* infection, with clinical resolution achieved in up to 81% of patients after a single infusion (van Nood et al., 2013). Furthermore, chronic inflammation and barrier disruption in IBD, driven by dysbiosis and pathogens including enterotoxigenic *B. fragilis* (ETBF), which activates Stat3 and promotes Th17-dependent carcinogenesis, significantly increase CRC risk (Wu et al., 2009; Grivennikov et al., 2012). In this context, FMT has been shown to induce clinical remission in 63% of IBD patients and facilitate discontinuation of immunosuppressants in 76%, thereby mitigating a key precancerous condition (Anderson et al., 2012; Wang ZK. et al., 2014). Additionally, FMT is being investigated as an adjunct to immunotherapy. Early-phase trials have demonstrated that it can restore anti-PD-1 responsiveness in refractory melanoma patients via remodeling both the gut microbiome and tumor microenvironment, highlighting its ability to enhance antitumor immunity (Baruch et al., 2021).

Looking forward, the development of lyophilized microbial consortia (e.g., *F. prausnitzii*-enriched capsules) offers a safer, a standardized,and more practical alternative to fresh stool for broader preventive application. However, several challenges remain, including limited long-term safety data and the risk of adverse events ranging from abdominal discomfort to serious infections or disease relapse. These concerns underscore the need for rigorous donor screening, standardized protocols, and well-controlled clinical trials before widely implemented in cancer prevention (Chen et al., 2019; Kazmierczak-Siedlecka et al., 2020).

### 4.3 Diet and Lifestyle Interventions

An accumulating body of evidence underscores the critical role of diet and lifestyle factors in directly shaping the composition and functional capacity of gut microbial ecosystems. The gut microbiome integrates these environmental cues with host physiology and metabolism, thereby modulating key biological processes involved in tumor initiation and progression (Song and Chan, 2019). In terms of dietary patterns, distinct nutritional profiles exert opposing effects on carcinogenesis, largely mediated through microbial modulation.

#### 4.3.1 Pro-carcinogenic diets

The Western dietary pattern, characterized by high consumption of saturated fats, refined sugars, and processed meats along with inadequate fiber intake, has emerged as a major risk factor for cancer development (Cordain et al., 2005). Increasing quantitative studies have demonstrated that continuous overconsumption of red meat and ultra-processed foods (UPFs) constitutes a key drivers of various tumor types (Ibragimova et al., 2021). Regular intake of red and processed meat, defined as unprocessed mammalian muscle such as beef and lamb along with chemically preserved products like bacon and salami, generates heterocyclic amines during high-temperature cooking that form mutagenic DNA adducts (Carr et al., 2017). Consistent findings from prospective epidemiological studies and meta-analyses show that red and processed meat convincingly elevates CRC risk by 20%–30% (Aykan, 2015). Concurrently, UPFs, which are industrial formulations containing five or more synthetic additives such as emulsifiers, artificial sweeteners, and hydrogenated fats, have been linked to increased cancer risk. A recent cohort study in the French population reported that a 10% increase indietary UPFs proportion was associated with an over 10% increase in the risk of overall cancer and breast cancer (Fiolet et al., 2018). Furthermore, high UPF intake has been positively linked to colorectal adenomas, particularly advanced and proximal lesions, with a pronounced dose-dependent effect observed among smokers (Fliss-Isakov et al., 2020).

#### 4.3.2 Protective dietary patterns

In contrast, several protective dietary patterns modulate the gut microbiota to suppress tumorigenesis.

Dietary fiber is a major substrate for microbial fermentation, producing beneficial short-chain fatty acids (SCFAs) such as butyrate, acetate, and propionate (So et al., 2018). Higher fiber intake enriches beneficial bacteria including *Lactobacillus spp*. and butyrate producing genera such as *Bifidobacterium, Roseburia*, and *Eubacterium*, thereby enhancing SCFA production (Holscher et al., 2015). Crucially, dietary fiber protects against colorectal tumorigenesis in a microbiota- and butyrate-dependent manner, primarily through histone deacetylase inhibition. This protective effect requires both fiber and butyrate-producing bacteria, as demonstrated by the synergistic reduction in tumor incidence when a high-fiber diet is combined with *Butyrivibrio fibrisolvens* supplementation reducing (Donohoe et al., 2014). Beyond direct epigenetic modulation, SCFAs contribute to immune regulation by promoting the differentiation and function of regulatory T cells (Treg), thereby suppressing inflammation and enhancing antitumor immunity. Butyrate and propionate induce Treg generation through histone deacetylase inhibition and GPR109a activation (Singh et al., 2014). The presence of SCFA-producing bacteria is linked to improved responses to immunotherapy, underscoring the role of fiber and microbial metabolites in modulating tumor immunity (Matson et al., 2018; Gopalakrishnan et al., 2018). Collectively, these findings demonstrate that dietary fiber influences colorectal cancer prevention through microbial production of SCFAs and immunomodulatory effects.

Beyond fiber, specific plant-derived polysaccharides from sources such as *Ganoderma lucidum*, *Zizyphus jujuba*, apple, and *Dendrobium officinale* have demonstrated profound anti-tumor effects. Mechanistically, these polysaccharides restore microbial balance and enhance SCFA production (Ji et al., 2019; Li et al., 2020), strengthen gut barrier integrity (Guo et al., 2021; Liang et al., 2019), inhibit pro-carcinogenic signaling pathways like Wnt/β-catenin, and potentiate anti-tumor immunity by improving the metabolic fitness while reducing exhaustion of cytotoxic T cells within the tumor microenvironment. This collective evidence highlights the role of these polysaccharides in modulating the gut microbiota-metabolite-immune axis for cancer prevention.

Marine omega-3 polyunsaturated fatty acids (PUFAs) also exhibit protective effects. Meta-analysis of biospecimen studies indicates that higher tissue levels of long-chain n-3 PUFAs, particularly EPA and DHA, are associated with a reduced risk of colorectal cancer (Yang et al., 2014). A large prospective cohort study further suggested a potential complex relationship with CRC subtypes and a possible latency period for the protective effect in men (Song et al., 2014). Importantly, higher intake of marine ω-3 PUFAs following CRC diagnosis has been associated with lower CRC-specific mortality, with incremental increases conferring additional survival benefits (Song et al., 2017). The anti-tumor mechanisms may extend beyond anti-inflammation; for example, a clinical intervention study using EPA-free fatty acid in CRC patients with liver metastases demonstrated anti-angiogenic activity in tumor tissue and a remarkable overall survival benefit despite similar early recurrence rates (Cockbain et al., 2014).

#### 4.3.3 Physical exercise

Exercise increases gut microbiota diversity (e.g., *Faecalibacterium, Akkermansia*) and enriches butyrate-producing taxa (e.g., *Roseburia*, *Eubacterium*) (Clarke et al., 2014; Bressa et al., 2017). This elevates SCFA production, particularly butyrate (Allen et al., 2018), which suppresses inflammation via HDAC inhibition and Treg differentiation, thereby reducing pro-inflammatory cytokines such as IL-17 (Levy et al., 2017; Zhang et al., 2019). Concurrently, butyrate strengthens gut barrier integrity via AMPK-dependent tight junction repair and mucin synthesis (Takiishi et al., 2017), while also promoting anticancer immunity through stimulating cytotoxic T-cell activation and inducing apoptosis in transformed cells. Collectively, these microbial and immunometabolic shifts contribute to reduced cancer risk.

## 5 Conclusion and future perspectives

As the ancient wisdom reminds us, “to prevent is better than to cure”. This review has synthesized compelling evidence that the gut microbiota stands as a pivotal modifier of cancer risk, orchestrating its effects through multifaceted mechanisms including modulation of the immune system, maintenance of epithelial barrier integrity, and detoxification of carcinogens (Bhatt et al., 2017). These common findings emphasize that preserving the probiotic nature of the symbiotic microbial community is fundamental to suppressing chronic inflammation, neutralizing genotoxic damage, and thereby preventing tumor initiation and progression (Bose and Mukherjee, 2019).

Interventions targeting the gut microbiome, such as probiotics, prebiotics, synbiotics, FMT, and dietary modifications, have demonstrated considerable promise in preclinical models by reinforcing protective host barriers and mitigating oncogenic pathways. However, translating these compelling experimental findings into tangible clinical applications for cancer prevention remains a formidable challenge.

First, bridging the “translational valley” between preclinical models and human application is imperative. The striking efficacy of probiotics and synbiotics in rodent models of carcinogenesis (Table 1) has not been consistently mirrored in human trials. This disconnect likely stems from profound inter-species differences in gut microbiome composition and host immunity, as well as the staggering inter-individual variability of the human microbiome, which is shaped by genetics, long-term diet, and environmental exposures (Tsilimigras et al., 2017).

**TABLE 1:** Summary of published studies on the potential use of probiotic, prebiotic, and symbiotic in cancer prevention.

Model used	Probiotic strains used	Major findings	Mechanistic exploration	Ref	Year
DMH-induced C57BL/6 mice	*Lactobacillus acidophilus, Lactobacillus paracasei, B. lactis, B. bifidum*	Inhibited NF-κB; reduced mucin depletion; increased Ki-67; reduced body weight	Modulation of TLR/NF-κB pathway; altered cytokine profile	Reis et al. (2024)	2023
Caco-2 cell line	Ethyl acetate extracts of *Lactobacillus plantarum* ATCC 14917 and *Lactobacillus rhamnosus* ATCC 7469	Reduced viability of Caco-2 cells (67%–71%); no effect on HUVEC cells	Activation of intrinsic apoptosis pathway; increased caspase-3 and -9 activity; downregulation of *bcl-2*, *bcl-xl*; upregulation of *bak*, *bad*, *bax*	Amin et al. (2023)	2023
C57BL/6 mouse model	*Lactobacillus plantarum* L168/Indole-3-lactic acid (ILA)	Ameliorated intestinal inflammation, tumor growth, and gut dysbiosis	Epigenetic regulation of CD8^+^ T cell immunity; enhanced H3K27ac binding at IL12a enhancer; reduced Saa3 expression via chromatin accessibility changes	Zhang et al. (2023)	2023
DMH-induced Wistar rat model	Vitamin D3; *Lactobacillus gasseri*; *Bifidobacterium bifidum*	Reduced ACF and AC counts in all treatment approaches (simultaneous, pre-, post-treatment); synergistic effect in pre-treatment; altered fecal microbiota	Modulation of Nrf2, GST, COX2, iNOS, β-catenin, PCNA expression; increased antioxidant response; anti-proliferative and anti-inflammatory effects; microbiota modulation ↑*Lactobacillus*, *Akkermansia*)	de Oliveira et al. (2023)	2023
AOM/DSS-induced CRC mouse model	*Lacticaseibacillus rhamnosus* LS8	Inhibited tumor formation; enhanced gut barrier; increased SCFAs; decreased LPS and pro-inflammatory cytokines	Regulated gut microbiota (↑ beneficial bacteria, e.g., *Faecalibaculum*, *Akkermansia*); inhibited TLR4/NF-κB pathway; strengthened tight junctions (ZO-1, occludin, claudin-1)	Wang et al. (2022a)	2022
AOM/DSS-induced CA-CRC mouse model	*Lactobacillus coryniformis* MXJ32	Reduced tumor number and diameter; improved gut barrier; decreased inflammation	Upregulated tight junction proteins; modulated gut microbiota (↑ beneficial bacteria, e.g., *Lactobacillus*, *Bifidobacterium*; ↓ harmful bacteria, e.g., *Desulfovibrio*, *Helicobacter*)	Wang et al. (2022b)	2022
AOM/DSS-induced mouse model	*Lactobacillus rhamnosus* Probio-M9	Reduced inflammation, tumor number and size; improved stool consistency; increased gut microbiota diversity	Suppression of p-Akt and p-STAT3; reduction in M1/M2 macrophages; restoration of beneficial bacteria (*Akkermansia*, *Bifidobacterium*); modulation of microbial metabolism	Xu et al. (2021)	2021
DMH-induced BALB/c mouse model	*Lactobacillus paracasei DTA81*	Reduced hepatic oxidative stress (MDA, carbonyl protein); decreased colonic IL-6, IL-17; increased acetic acid and total SCFAs; enriched Ruminiclostridium	Antioxidant activity; anti-inflammatory effects; gut microbiota modulation; SCFA production	da Silva Duarte et al. (2020)	2020
DMH-induced F344 rat model	*Lactobacillus salivarius* Ren	Suppressed tumor formation; induced apoptosis and cell cycle arrest	Inhibition of AKT phosphorylation; downregulation of cyclinD1 and COX-2	Dong et al. (2020)	2020
DMH-induced rat model	*Lactobacillus paracasei* X12	Reduced tumor incidence (∼66%), volume, and multiplicity; prevented severe weight loss; increased apoptotic index	Upregulation of pro-apoptotic genes (Bax, Caspase-3, Caspase-9); downregulation of anti-apoptotic genes (Bcl-2, Jak-1, Akt-1); induction of apoptosis via intrinsic and extrinsic pathways; inhibition of cell proliferation	Mousavi Jam et al. (2020)	2020
DMH/DSS-induced rat model	Djulis + *Lactobacillus acidophilus*	Reduced total ACF, SIM-ACF, and MDF; downregulated PCNA and COX-2; regulated apoptosis-related proteins	Regulation of proliferative, inflammatory, and apoptotic pathways; enhanced fecal *L. acidophilus* count; reduced cecal pH	Lee et al. (2019)	2020
DMH-induced mouse model	Inulin + *Lactobacillus casei*	Reduced CEA levels and ACF number; increased p-JNK-1; decreased β-catenin and p-GSK3β	Modulation of JNK-1/β-catenin signaling pathway; enrichment of beneficial genera (*Akkermansia*, *Turicibacter*)	Ali et al. (2019)	2019
DMH-induced rat model	*Lactobacillus rhamnosus* GG, *Lactobacillus acidophilus* + Celecoxib	Reduced tumor burden and multiplicity; upregulated Bax and p53; downregulated Bcl-2 and K-ras	Enhanced apoptosis; modulation of pro-/anti-apoptotic genes; gut microenvironment modification	Sharaf et al. (2018)	2018
DMH-induced rat model	*Lactobacillus plantarum* (AdF10), *Lactobacillus rhamnosus* GG	Increased antioxidant enzymes; normalized apoptosis-related protein expression	Reduction in oxidative stress; regulation of p53, Bax, Bcl-2, caspases; enhancement of antioxidant defense	Walia et al. (2018)	2018
AOM/DSS-induced CRC mouse model	*Lactobacillus casei* BL23	Reduced tumor incidence and proliferation; decreased histological damage	Downregulated IL-22; upregulated pro-apoptotic genes (caspase-7, caspase-9, Bik); modulated gut microbiota	Jacouton et al. (2017)	2017
DMH-induced BALB/c mice model	*Lactobacillus casei* ATCC 393	Reduced ACF; decreased putrescine; downregulated ODC	Maintenance of polyamine metabolism; antimutagenic effect	Irecta-Najera et al. (2017)	2017
DMH-induced Sprague-Dawley rat model	*Lactobacillus rhamnosus*GG CCMCC 1.2134	Reduced tumor incidence, multiplicity, volume; induced apoptosis	Downregulation of NF-κB, COX-2, TNF-α, Bcl-2; upregulation of Bax, Casp3, p53, β-catenin	Gamallat et al. (2016)	2016
DMH-induced rat model	*L. acidophilus, B. bifidum, B. infantum* + oligofructose/maltodextrin	Reduced tumor incidence, multiplicity, and volume; altered gut microbiota	Enhanced mucosal barrier (MUC2, ZO-1, occludin); increased TLR2; decreased TLR4, COX-2, caspase-3, β-catenin	Kuugbee et al. (2016)	2016
*H. pylori*-infected mouse model	*Lactobacillus plantarum* ZDY 2013	Prevented gastric inflammation and microbiota alteration induced by *H. pylori*	Suppressed Th1/Th17 response; inhibited pro-inflammatory cytokines; modulated gastric microbiota (↑ Firmicutes/Bacteroidetes; ↓ Proteobacteria)	Pan et al. (2016)	2016
Apc^Min/+^ mouse model	Microencapsulated *Lactobacillus acidophilus* in yogurt	Reduced total intestinal tumor number by 44% (4.5 vs. 2.5 tumors/mouse); Reduced proliferation (Ki-67); Altered immune cell infiltration	Increased CD8^+^ T cells in tissue; Modulated apoptosis (cleaved caspase-3); Potential immunomodulation and anti-proliferative effects	Urbanska et al. (2016)	2016
CT26 tumour-bearing mouse model	*Lactobacillus plantarum*	Inhibited CT26 tumor growth, prolonged survival, increased NK and CD8^+^ T cell infiltration in tumors	Enhanced NK and CD8^+^ T cell activity; promotion of Th1 response; upregulation of IFN-γ; dendritic cell maturation	Hu et al. (2015)	2015
Gallus gallus (chicks) model	*Lactobacillus rhamnosus* Vc	Reduced MNNG-induced genotoxicity (69%) and mutagenicity (61%) *in vitro*; decreased GST activity *in vivo*; attenuated colon and liver damage	Detoxification of MNNG to less toxic metabolites; reduction in inflammation	Pithva et al. (2015)	2015
DMH-induced rat model	*Lactobacillus salivarius*	Decreased cancer incidence (87.5%–25%); Counteracted DMH-induced gut microbiota dysbiosis	Increased *Prevotella*; Decreased *Ruminococcus* sp., *Clostridiales*, *Bacteroides dorei*; Reversion of gut microbiota towards a healthy state	Zhang et al. (2015)	2015
LS174T cells; mouse colonic epithelium	p40 (a protein derived from *Lactobacillus rhamnosus* GG)	Upregulated *Muc2* gene expression and mucin production; thickened colonic mucus layer	Activation of EGFR and Akt; mucin production via EGFR transactivation	Wang et al. (2014a)	2014
DMH-induced F344 rat model	*Lactobacillus salivarius* Ren	Reduced ACF number (∼40%) and epithelial proliferation; improved microbiota structure	Increased SCFA (butyrate); decreased azoreductase activity; modulation of gut microbiota (increased *Prevotella*, decreased *Bacillus*)	Zhu et al. (2014)	2014
PhIP-treated mouse model	*Lactobacillus rhamnosus* IMC501	Reduced PhIP-induced DNA damage in colonocytes; increased fecal lactobacilli; decreased β-glucuronidase and N-acetyl-β-glucosaminidase activities	Binding/sequestration of genotoxins; modulation of gut microbiota and enzyme activities; reduction in genotoxic metabolites	Dominici et al. (2014)	2014
DMH-induced Swiss mouse model	*Lactobacillus delbrueckii* UFV-H2b20, *B.animalis* var. *lactis* Bb12, *S. boulardii*	*L. delbrueckii* and *B. animalis* alone reduced total ACF; no effect from combination or *S. boulardii*	Strain-specific protection; reduction mainly in small ACF (≤3 crypts); possible modulation of microbiota or immune response	Liboredo et al. (2013)	2013
Cyclic DSS-treated rat model	Blueberry husks + Probiotic mixture (*B. infantis, Lactobacillus gasseri, Lactobacillus plantarum*)	Reduced DAI, number of colonic ulcers and dysplastic lesions; decreased fecal Enterobacteriaceae; increased lactobacilli; mitigated liver injury	Altered SCFA profiles (decreased butyrate, increased acetate); reduced bacterial translocation; modulation of gut microbiota	Hakansson et al. (2012)	2012
DMH-induced ICR mouse model	*Bifidobacterium longum*, *Lactobacillus gasseri*	Inhibited ACF formation; reduced colon tumor multiplicity and size	Suppressed colonic mucosa cellular proliferation; enhanced phagocytic activity of macrophages	Foo et al. (2011)	2011
PhIP-induced ACF rat model; DMH-induced tumors rasH2 mice model	Yogurt (*Lactobacillus. delbrueckii* subsp. *bulgaricus* 2038 + *S. salivarius* subsp. *thermophilus* 1131)	Reduced ACF and ACs in rats; reduced number of colorectal tumors in rasH2 mice	Possible immunomodulation; antioxidant activity; alteration of gut microbiota or mutagen sequestration	Narushima et al. (2010)	2010
DMH-induced rat model	*Lactobacillus acidophilus*, *Lactobacillus casei*, *Lactobacillus lactis* biovar. *diacetylactis*	Significantly reduced DNA damage (54.7% vs. 88.1% in DMH control) and tumor incidence in colon	Reduction in free radicals, prevention of DNA adduct formation; enhancement of antioxidant activity	Kumar et al. (2010)	2009
AOM-induced Fisher rat model	EPS-producing and non-EPS-producing lactic cultures (e.g., *S. thermophilus*, *L. bulgaricus*, *L. lactis*)	Reduced colon tumor incidence and multiplicity with some strains; no correlation between EPS properties and chemoprevention	Reduction in COX-2 activity; possible interaction of EPS/metabolites with milk components	Purohit et al. (2009)	2009
WD-induced CAC mouse model	Probiotic VSL#3 + Metformin	Combination significantly ameliorated colitis and tumor growth; reduced macrophage infiltration; maintained epithelial integrity	Enhanced late apoptosis via inhibition of cyclin D1 and Bcl-2; activation of pro-apoptotic ERK and AMPK pathways	Chung et al. (2017)	2016
AOM/DSS mouse model	Probiotic VSL#3 + Balsalazide	Significantly reduced total tumor number and macrophage infiltration; decreased F4/80+ macrophages in tumor stroma	Suppression of IL-6/STAT3 pathway; decreased MIP-1β, MCP-1, IL-6, IL-10; downregulation of BCL2, upregulation of BAX and p-ERK.	Do et al. (2016)	2016
DSS-induced chronic colitis mouse model	Probiotic VSL#3	Attenuated DAI, reduced dysplasia and adenocarcinoma incidence; no carcinoma in concurrent VSL#3 group	Reduced PCNA labeling index; decreased TNF-α, IL-1β, IL-6, COX-2; increased IL-10	Talero et al. (2015)	2015
AOM/III0^−/−^ mouse model	Probiotic VSL#3	Did not reduce tumorigenesis or inflammation; Enhanced tumor penetrance, multiplicity, and invasion in one cohort	Altered mucosal microbiota composition (16-fold decrease in a *Clostridium* taxon); Depletion of specific bacteria potentially mediated increased tumorigenesis	Arthur et al. (2013)	2013
AOM/DSS and H.typhlonius/IL-10^−/−^ mouse models	Probiotic VSL#3	Reduced disease activity, tumor number, and size; decreased adenoma/adenocarcinoma formation	Upregulation of TNF-α, PPARγ, angiotatin; downregulation of COX-2; increased IL-17^+^ CD4^+^ T cells in MLN and Tregs in lamina propria	Bassaganya-Riera et al. (2012)	2012
TNBS-induced rat model	Probiotic VSL#3	Delayed transition from inflammation to dysplasia; reduced macroscopic damage; no carcinoma in VSL#3 group vs. 29% in controls	Increased angiotatin and VDR expression; decreased alkaline phosphatase; modulation of gut microbiota richness/diversity	Appleyard et al. (2011)	2011
Human colon cancer cell lines	Conditioned medium from *L. lactis* HkyuLL 10	Inhibited viability and proliferation of CRC cells; no effect on normal colon cells	Secreted proteins >100 kDa (e.g., α-mannosidase) induced apoptosis and inhibited proliferation	Su et al. (2024)	2024
CRC xenograft mouse model	α-mannosidase (from *L. lactis* HkyuLL 10)	Suppressed tumor growth in HCT116 and HT29 xenografts	Decreased Ki-67+ cells; increased TUNEL+ cells; induction of apoptosis and inhibition of proliferation	Su et al. (2024)	2024
Germ-free AOM/DSS mouse model	*L. lactis* HkyuLL 10	Reduced tumor number and load; increased apoptosis	Demonstrated direct antitumor effect independent of microbiota modulation	Su et al. (2024)	2024
Sprague-Dawley rat model/NU/NU nude mouse model	Microencapsulated *Lactiplantibacillus plantarum* LAB12	Targeted release in intestines; reduced tumor volume (−98.87%) and weight (−89.27%)	Upregulation of p53 and caspase-3; downregulation of COX-2, VEGF, PECAM-1; induction of apoptosis and anti-angiogenesis	Fareez et al. (2024)	2022
DMH-induced rat model	Isomatooligosaccharides (IMOs)	Inhibited ACF formation (29.4% reduction); reduced serum inflammatory cytokines (TNF-α, IL-1β, IL-6, IL-8); improved gut barrier function; modulated gut microbiota	Increased abundance of *Lactobacillus*; decreased *Fusobacterium*; reduced fecal β-glucuronidase and β-glucosidase; decreased bile acids and amino acids; anti-inflammatory and barrier-protective effects	Chen et al. (2022)	2022
DMH-induced Wistar rat model	Kefir milk (a probiotic fermented milk produced from kefir grains)	Reduced tumor incidence by 100% (KNL) and 71.43% (KSL); decreased IL-1β, IL-6, TNF-α, NO; increased Romboutsia	Anti-inflammatory cytokine reduction; gut microbiota modulation; increased beneficial bacteria	Guiomar de Almeida Brasiel et al. (2022)	2022
DMH-induced BALB/c mouse model	Sphingomyelin (0.05%) + *Lacticaseibacillus casei* + *Bifidobacterium bifidum*	Reduced number of aberrant crypt foci (ACF); improved galactose uptake in enterocyte brush-border membrane vesicles	Enhanced intestinal physiological function; possible synergy between sphingomyelin and probiotics	Marzo et al. (2022)	2022
AOM-induced rat model	Inulin + *Bifidobacterium longum*	Synergistic reduction in ACF, especially those with high multiplicity	Enhanced gut fermentation; production of SCFAs; modulation of bacterial enzymes	Pool-Zobel et al. (2002)	2002
AOM/DSS-induced mouse model	*Ganoderma lucidum* polysaccharide (GLP)	Alleviated colitis and tumorigenesis; improved gut microbiota; increased SCFAs; enhanced gut barrier	Inhibited TLR4/MyD88/NF-κB signaling; reduced inflammation and macrophage infiltration	Guo et al. (2021)	2021
Apc^Min/+^ and AOM-injected mouse model	*Streptococcus thermophilus*	Reduced tumor formation; inhibited proliferation of CRC cells; induced cell cycle arrest and apoptosis	Secretion of β-galactosidase; production of galactose; activation of oxidative phosphorylation; inhibition of Hippo pathway	Li et al. (2021b)	2021
DMH-induced rat model	*Clostridium butyricum*, *Bacillus subtilis*	Inhibited CRC cell proliferation, induced apoptosis and cell cycle arrest; reduced tumor incidence and size in DMH-induced mice	Downregulation of TLR4–MyD88–NF-κB pathway; reduction in Th2/Th17 responses; upregulation of P21; decreased inflammation	Chen et al. (2015)	2015
AOM-induced Sprague-Dawley rat model	*Bifidobacterium lactis* + Resistant starch (synbiotic)	Synbiotic significantly reduced neoplasm incidence and multiplicity; RS alone showed a trend; probiotic alone ineffective	Increased SCFA production; reduced epithelial cell proliferation; no change in spontaneous apoptosis	Le Leu et al. (2010)	2010
AOM-induced Sprague-Dawley rat model	*Bifidobacterium lactis* + Resistant starch (synbiotic)	Synbiotic combination significantly facilitated acute apoptotic response to genotoxic carcinogen in distal colon; neither probiotic nor prebiotic alone had an effect	RS acts as metabolic substrate, creating conditions for B. lactis to exert pro-apoptotic effects; increased SCFA production; no change in spontaneous apoptosis	Le Leu et al. (2005)	2005
Apc^Min/+^ mouse model	*Saccharomyces boulardii*	Inhibited intestinal tumor growth and dysplasia; reduced tumor number and size	Inactivation of EGFR, HER-2, HER-3, IGF-1R signaling; inhibition of cell proliferation; induction of apoptosis	Chen et al. (2009)	2009
DMH-induced F344 rat model	*Bacillus polyfermenticus*	Reduced aberrant crypt foci (ACF) by 50%; Decreased leukocytic DNA damage and plasma lipid peroxidation; Increased plasma antioxidant potential	Attenuation of DMH-induced genotoxicity and oxidative stress; Enhancement of total antioxidant capacity	Park et al. (2007)	2007
DMH-induced rat model	*Bacillus polyfermenticus*	Strong adherence to Caco-2 cells; Inhibited colon cancer cell growth (dose-dependent); Reduced DMH-induced aberrant crypts by 40%	High cell surface hydrophobicity and agglutination; Proposed mechanisms include antimicrobial effects and potential induction of apoptosis	Lee et al. (2007)	2006
IL-10^−/−^ mouse model	*Enterococcus faecalis, Escherichia coli*, *Pseudomonas fluorescens*	*E. faecalis* and *E. coli* induced region-specific colitis; P. fluorescens did not	Bacterial antigen-specific CD4^+^ T-cell responses; differential cytokine profiles; regional immune activation	Kim et al. (2005)	2005
AOM-induced F344 rat model	Inulin enriched with oligofructose (Synergy1®); *L. rhamnosus* GG; *B. lactis* Bb12; symbiotic combination	Synergy1 significantly reduced colorectal tumor number; probiotics slightly reduced malignant tumors	Increased SCFA (butyrate); reduced proliferation; altered expression of GST-P, iNOS, COX-2; increased apoptosis in normal mucosa	Femia et al. (2002)	2002
ApcMin/+ mouse model	Oligofructose	Reduced colon tumors; enhanced gut-associated lymphoid tissue (GALT)	Immunomodulation; possible prebiotic effect on gut immunity	Pool-Zobel et al. (2002)	2002

Second, there is an imperative to move beyond microbial correlation and establish causative mechanisms. Although numerous associations have been documented between specific bacteria (e.g., *Bifidobacterium, Akkermansia*) and improved anti-tumor immunity, the precise molecular messenger, whether the SCFAs like butyrate, unique metabolites like indole-3-lactic acid, or bacterial components themselves, often remain elusive (Smith et al., 2013; Zhang et al., 2023). Future research must, therefore, integrate multi-omics technologies (metagenomics, meta transcriptomics, metabolomics) with gnotobiotic models and sophisticated *in vitro* systems like organoids to dissect the precise mechanistic links between specific microbial strains, their metabolites, and host anti-tumor pathways (Sun et al., 2025). This mechanistic clarity is the basis for identifying reliable targets.

Third, the identification of key microbial molecules and pathways naturally paves the way for precision prevention. Mechanistic insights can be translated into clinical practice through diagnostics that screen for these specific microbial genes or metabolite profiles as biomarkers of increased cancer risk, enabling early detection of high-risk individuals. Personalized prevention strategies could include FMT from optimally screened donors to restore microbial balance or the development of engineered microbial consortia and “postbiotic” formulations, defined microbial metabolites that bypass the challenges of live bacterial delivery while directly exerting protective effect (Chen et al., 2019). Achieving this vision will require concerted, consortium-level efforts to standardize microbiome research methodologies, from sample collection to bioinformatics analysis, to ensure reproducibility, data comparability and accelerated discovery.

Finally, the scope of the gut microbiome’s influence demands a systemic perspective. Microbial metabolites are not confined to the intestinal lumen but circulate throughout the body, influencing distant organs. The gut-lung axis, for instance, where gut microbiota-derived SCFAs modulate immune responses in the lung, illustrates the potential for gut-focused interventions to prevent extraintestinal malignancies (Hanus et al., 2021). Similarly, microbiota-modified bile acids regulate hepatic NKT cell accumulation, shaping immune surveillance in HCC (Ma et al., 2018). These findings reveal a paradigm shift: modulating the gut microbiome is not merely a strategy for CRC prevention but a gateway to influencing systemic oncogenic risk and therapeutic efficacy across cancer types.

In summary, while our understanding of the gut microbiota’s role in cancer prevention is still evolving, the path forward is clear. By harnessing emerging technologies, fostering interdisciplinary collaboration, and prioritizing human trials, we can begin to strategically manipulate this intricate internal ecosystem. The ultimate goal is to integrate microbiome modulation into the framework of precision medicine, paving the way for novel, effective, and personalized strategies to reduce the global burden of cancer.

## References

[B1] Albillos A. de Gottardi A. Rescigno M. (2020). The gut-liver axis in liver disease: pathophysiological basis for therapy. J. Hepatol. 72 (3), 558–577. 10.1016/j.jhep.2019.10.003 31622696

[B2] Ali M. S. Hussein R. M. Gaber Y. Hammam O. A. Kandeil M. A. (2019). Modulation of JNK-1/beta-catenin signaling by Lactobacillus casei, inulin and their combination in 1,2-dimethylhydrazine-induced colon cancer in mice. RSC Adv. 9 (50), 29368–29383. 10.1039/c9ra04388h 35528422 PMC9071812

[B3] Allen J. M. Mailing L. J. Niemiro G. M. Moore R. Cook M. D. White B. A. (2018). Exercise alters gut microbiota composition and function in lean and Obese humans. Med. Sci. Sports Exerc 50 (4), 747–757. 10.1249/MSS.0000000000001495 29166320

[B4] Amin M. Navidifar T. Saeb S. Barzegari E. Jamalan M. (2023). Tumor-targeted induction of intrinsic apoptosis in colon cancer cells by Lactobacillus plantarum and Lactobacillus rhamnosus strains. Mol. Biol. Rep. 50 (6), 5345–5354. 10.1007/s11033-023-08445-x 37155013

[B5] Anderson J. L. Edney R. J. Whelan K. (2012). Systematic review: faecal microbiota transplantation in the management of inflammatory bowel disease. Aliment. Pharmacol. Ther. 36 (6), 503–516. 10.1111/j.1365-2036.2012.05220.x 22827693

[B6] Appleyard C. B. Cruz M. L. Isidro A. A. Arthur J. C. Jobin C. De Simone C. (2011). Pretreatment with the probiotic VSL#3 delays transition from inflammation to dysplasia in a rat model of colitis-associated cancer. Am. J. Physiol. Gastrointest. Liver Physiol. 301 (6), G1004–G1013. 10.1152/ajpgi.00167.2011 21903764 PMC3233787

[B7] Arnone A. A. Cook K. L. (2022). Gut and breast microbiota as endocrine regulators of hormone receptor-positive breast cancer risk and therapy response. Endocrinology 164 (1), bqac177. 10.1210/endocr/bqac177 36282876 PMC9923803

[B8] Arpaia N. Campbell C. Fan X. Dikiy S. van der Veeken J. deRoos P. (2013). Metabolites produced by commensal bacteria promote peripheral regulatory T-cell generation. Nature 504 (7480), 451–455. 10.1038/nature12726 24226773 PMC3869884

[B9] Arthur J. C. Gharaibeh R. Z. Uronis J. M. Perez-Chanona E. Sha W. Tomkovich S. (2013). VSL#3 probiotic modifies mucosal microbial composition but does not reduce colitis-associated colorectal cancer. Sci. Rep. 3, 2868. 10.1038/srep02868 24100376 PMC3792409

[B10] Atarashi K. Tanoue T. Shima T. Imaoka A. Kuwahara T. Momose Y. (2011). Induction of colonic regulatory T cells by indigenous clostridium species. Science. 331 (6015), 337–341. 10.1126/science.1198469 21205640 PMC3969237

[B11] Aykan N. F. (2015). Red meat and colorectal cancer. Oncol. Rev. 9 (1), 288. 10.4081/oncol.2015.288 26779313 PMC4698595

[B12] Azcarate-Peril M. A. Sikes M. Bruno-Barcena J. M. (2011). The intestinal microbiota, gastrointestinal environment and colorectal cancer: a putative role for probiotics in prevention of colorectal cancer? Am. J. Physiol. Gastrointest. Liver Physiol. 301 (3), G401–G424. 10.1152/ajpgi.00110.2011 21700901 PMC3774253

[B13] Backhed F. Ley R. E. Sonnenburg J. L. Peterson D. A. Gordon J. I. (2005). Host-bacterial mutualism in the human intestine. Science 307 (5717), 1915–1920. 10.1126/science.1104816 15790844

[B14] Baruch E. N. Youngster I. Ben-Betzalel G. Ortenberg R. Lahat A. Katz L. (2021). Fecal microbiota transplant promotes response in immunotherapy-refractory melanoma patients. Science. 371 (6529), 602–609. 10.1126/science.abb5920 33303685

[B15] Bassaganya-Riera J. Viladomiu M. Pedragosa M. De Simone C. Hontecillas R. (2012). Immunoregulatory mechanisms underlying prevention of colitis-associated colorectal cancer by probiotic bacteria. PLoS One 7 (4), e34676. 10.1371/journal.pone.0034676 22511958 PMC3325233

[B16] Bharti R. Grimm D. G. (2021). Current challenges and best-practice protocols for microbiome analysis. Brief. Bioinform 22 (1), 178–193. 10.1093/bib/bbz155 31848574 PMC7820839

[B17] Bhatt A. P. Redinbo M. R. Bultman S. J. (2017). The role of the microbiome in cancer development and therapy. CA Cancer J. Clin. 67 (4), 326–344. 10.3322/caac.21398 28481406 PMC5530583

[B18] Bose M. Mukherjee P. (2019). Role of microbiome in modulating immune responses in cancer. Mediat. Inflamm. 2019, 4107917. 10.1155/2019/4107917 31308831 PMC6594313

[B19] Bressa C. Bailen-Andrino M. Perez-Santiago J. Gonzalez-Soltero R. Perez M. Montalvo-Lominchar M. G. (2017). Differences in gut microbiota profile between women with active lifestyle and sedentary women. PLoS One 12 (2), e0171352. 10.1371/journal.pone.0171352 28187199 PMC5302835

[B20] Bruno-Barcena J. M. Azcarate-Peril M. A. (2015). Galacto-oligosaccharides and colorectal cancer: feeding our intestinal probiome. J. Funct. Foods 12, 92–108. 10.1016/j.jff.2014.10.029 25584074 PMC4288025

[B21] Carr P. R. Jansen L. Bienert S. Roth W. Herpel E. Kloor M. (2017). Associations of red and processed meat intake with major molecular pathological features of colorectal cancer. Eur. J. Epidemiol. 32 (5), 409–418. 10.1007/s10654-017-0275-6 28646407

[B22] Chen X. Fruehauf J. Goldsmith J. D. Xu H. Katchar K. K. Koon H. W. (2009). Saccharomyces boulardii inhibits EGF receptor signaling and intestinal tumor growth in apc(min) mice. Gastroenterology 137 (3), 914–923. 10.1053/j.gastro.2009.05.050 19482027 PMC2777664

[B23] Chen Y. Segers S. Blaser M. J. (2013). Association between *Helicobacter pylori* and mortality in the NHANES III study. Gut 62 (9), 1262–1269. 10.1136/gutjnl-2012-303018 23303440 PMC3834579

[B24] Chen Z. F. Ai L. Y. Wang J. L. Ren L. L. Yu Y. N. Xu J. (2015). Probiotics Clostridium butyricum and Bacillus subtilis ameliorate intestinal tumorigenesis. Future Microbiol. 10 (9), 1433–1445. 10.2217/fmb.15.66 26346930

[B25] Chen D. Wu J. Jin D. Wang B. Cao H. (2019). Fecal microbiota transplantation in cancer management: current status and perspectives. Int. J. Cancer 145 (8), 2021–2031. 10.1002/ijc.32003 30458058 PMC6767494

[B26] Chen X. Li S. Lin C. Zhang Z. Liu X. Wang C. (2022). Isomaltooligosaccharides inhibit early colorectal carcinogenesis in a 1,2-dimethylhydrazine-induced rat model. Front. Nutr. 9, 995126. 10.3389/fnut.2022.995126 36185671 PMC9521046

[B27] Chu H. Mazmanian S. K. (2013). Innate immune recognition of the microbiota promotes host-microbial symbiosis. Nat. Immunol. 14 (7), 668–675. 10.1038/ni.2635 23778794 PMC4109969

[B28] Chung H. Kasper D. L. (2010). Microbiota-stimulated immune mechanisms to maintain gut homeostasis. Curr. Opin. Immunol. 22 (4), 455–460. 10.1016/j.coi.2010.06.008 20656465

[B29] Chung E. J. Do E. J. Kim S. Y. Cho E. A. Kim D. H. Pak S. (2017). Combination of metformin and VSL#3 additively suppresses western-style diet induced colon cancer in mice. Eur. J. Pharmacol. 794, 1–7. 10.1016/j.ejphar.2016.11.012 27845068

[B30] Clarke S. F. Murphy E. F. O'Sullivan O. Lucey A. J. Humphreys M. Hogan A. (2014). Exercise and associated dietary extremes impact on gut microbial diversity. Gut 63 (12), 1913–1920. 10.1136/gutjnl-2013-306541 25021423

[B31] Cockbain A. J. Volpato M. Race A. D. Munarini A. Fazio C. Belluzzi A. (2014). Anticolorectal cancer activity of the omega-3 polyunsaturated fatty acid eicosapentaenoic acid. Gut 63 (11), 1760–1768. 10.1136/gutjnl-2013-306445 24470281

[B32] Coffelt S. B. Kersten K. Doornebal C. W. Weiden J. Vrijland K. Hau C. S. (2015). IL-17-producing gammadelta T cells and neutrophils conspire to promote breast cancer metastasis. Nature 522 (7556), 345–348. 10.1038/nature14282 25822788 PMC4475637

[B33] Coker O. O. Dai Z. Nie Y. Zhao G. Cao L. Nakatsu G. (2018). Mucosal microbiome dysbiosis in gastric carcinogenesis. Gut 67 (6), 1024–1032. 10.1136/gutjnl-2017-314281 28765474 PMC5969346

[B34] Collins S. L. Stine J. G. Bisanz J. E. Okafor C. D. Patterson A. D. (2023). Bile acids and the gut microbiota: metabolic interactions and impacts on disease. Nat. Rev. Microbiol. 21 (4), 236–247. 10.1038/s41579-022-00805-x 36253479 PMC12536349

[B35] Cordain L. Eaton S. B. Sebastian A. Mann N. Lindeberg S. Watkins B. A. (2005). Origins and evolution of the Western diet: health implications for the 21st century. Am. J. Clin. Nutr. 81 (2), 341–354. 10.1093/ajcn.81.2.341 15699220

[B36] Correa N. B. Peret Filho L. A. Penna F. J. Lima F. M. Nicoli J. R. (2005). A randomized formula controlled trial of Bifidobacterium lactis and Streptococcus thermophilus for prevention of antibiotic-associated diarrhea in infants. J. Clin. Gastroenterol. 39 (5), 385–389. 10.1097/01.mcg.0000159217.47419.5b 15815206

[B37] da Silva Duarte V. Dos Santos Cruz B. C. Tarrah A. Sousa Dias R. de Paula Dias Moreira L. Lemos Junior W. J. F. (2020). Chemoprevention of DMH-induced early Colon carcinogenesis in Male BALB/c mice by administration of Lactobacillus paracasei DTA81. Microorganisms 8 (12), 1994. 10.3390/microorganisms8121994 33327620 PMC7765108

[B38] de Oliveira C. S. Baptistella M. M. Siqueira A. P. Carvalho M. O. Ramos L. F. Souto B. S. (2023). Combination of vitamin D and probiotics inhibits chemically induced colorectal carcinogenesis in wistar rats. Life Sci. 322, 121617. 10.1016/j.lfs.2023.121617 37003542

[B39] Dheer R. Santaolalla R. Davies J. M. Lang J. K. Phillips M. C. Pastorini C. (2016). Intestinal epithelial toll-like receptor 4 signaling affects epithelial function and colonic microbiota and promotes a risk for transmissible colitis. Infect. Immun. 84 (3), 798–810. 10.1128/IAI.01374-15 26755160 PMC4771346

[B40] Do E. J. Hwang S. W. Kim S. Y. Ryu Y. M. Cho E. A. Chung E. J. (2016). Suppression of colitis-associated carcinogenesis through modulation of IL-6/STAT3 pathway by balsalazide and VSL#3. J. Gastroenterol. Hepatol. 31 (8), 1453–1461. 10.1111/jgh.13280 26711554

[B41] Dominici L. Villarini M. Trotta F. Federici E. Cenci G. Moretti M. (2014). Protective effects of probiotic Lactobacillus rhamnosus IMC501 in mice treated with PhIP. J. Microbiol. Biotechnol. 24 (3), 371–378. 10.4014/jmb.1309.09072 24346468

[B42] Dong Y. Zhu J. Zhang M. Ge S. Zhao L. (2020). Probiotic Lactobacillus salivarius ren prevent dimethylhydrazine-induced colorectal cancer through protein kinase B inhibition. Appl. Microbiol. Biotechnol. 104 (17), 7377–7389. 10.1007/s00253-020-10775-w 32666185

[B43] Donohoe D. R. Holley D. Collins L. B. Montgomery S. A. Whitmore A. C. Hillhouse A. (2014). A gnotobiotic mouse model demonstrates that dietary fiber protects against colorectal tumorigenesis in a microbiota- and butyrate-dependent manner. Cancer Discov. 4 (12), 1387–1397. 10.1158/2159-8290.CD-14-0501 25266735 PMC4258155

[B44] Dupraz L. Magniez A. Rolhion N. Richard M. L. Da Costa G. Touch S. (2021). Gut microbiota-derived short-chain fatty acids regulate IL-17 production by mouse and human intestinal gammadelta T cells. Cell Rep. 36 (1), 109332. 10.1016/j.celrep.2021.109332 34233192

[B45] El T. G. Garrett W. S. (2023). Bacteria in cancer initiation, promotion and progression. Nat. Rev. Cancer 23 (9), 600–618. 10.1038/s41568-023-00594-2 37400581

[B46] Ervin S. M. Redinbo M. R. (2020). The gut microbiota impact cancer etiology through “Phase IV Metabolism” of xenobiotics and endobiotics. Cancer Prev. Res. (Phila) 13 (8), 635–642. 10.1158/1940-6207.CAPR-20-0155 32611614 PMC7980665

[B47] Ervin S. M. Li H. Lim L. Roberts L. R. Liang X. Mani S. (2019). Gut microbial beta-glucuronidases reactivate estrogens as components of the estrobolome that reactivate estrogens. J. Biol. Chem. 294 (49), 18586–18599. 10.1074/jbc.RA119.010950 31636122 PMC6901331

[B48] Fareez I. M. Lim S. M. Ramasamy K. (2024). Chemoprevention by microencapsulated Lactiplantibacillus plantarum LAB12 against orthotopic colorectal cancer mice is associated with apoptosis and anti-angiogenesis. Probiotics Antimicrob. Proteins 16 (1), 99–112. 10.1007/s12602-022-10020-y 36508139

[B49] Femia A. P. Luceri C. Dolara P. Giannini A. Biggeri A. Salvadori M. (2002). Antitumorigenic activity of the prebiotic inulin enriched with oligofructose in combination with the probiotics Lactobacillus rhamnosus and Bifidobacterium lactis on azoxymethane-induced colon carcinogenesis in rats. Carcinogenesis 23 (11), 1953–1960. 10.1093/carcin/23.11.1953 12419846

[B50] Ferreira R. M. Pereira-Marques J. Pinto-Ribeiro I. Costa J. L. Carneiro F. Machado J. C. (2018). Gastric microbial community profiling reveals a dysbiotic cancer-associated microbiota. Gut 67 (2), 226–236. 10.1136/gutjnl-2017-314205 29102920 PMC5868293

[B51] Fiolet T. Srour B. Sellem L. Kesse-Guyot E. Alles B. Mejean C. (2018). Consumption of ultra-processed foods and cancer risk: results from NutriNet-Sante prospective cohort. BMJ 360, k322. 10.1136/bmj.k322 29444771 PMC5811844

[B52] Fiorucci S. Distrutti E. (2015). Bile acid-activated receptors, intestinal microbiota, and the treatment of metabolic disorders. Trends Mol. Med. 21 (11), 702–714. 10.1016/j.molmed.2015.09.001 26481828

[B53] Fliss-Isakov N. Zelber-Sagi S. Ivancovsky-Wajcman D. Shibolet O. Kariv R. (2020). Ultra-processed food intake and smoking interact in relation with colorectal adenomas. Nutrients 12 (11). 10.3390/nu12113507 33202603 PMC7698317

[B54] Flores R. Shi J. Fuhrman B. Xu X. Veenstra T. D. Gail M. H. (2012). Fecal microbial determinants of fecal and systemic estrogens and estrogen metabolites: a cross-sectional study. J. Transl. Med. 10, 253. 10.1186/1479-5876-10-253 23259758 PMC3552825

[B55] Foo N. P. Ou Yang H. Chiu H. H. Chan H. Y. Liao C. C. Yu C. K. (2011). Probiotics prevent the development of 1,2-dimethylhydrazine (DMH)-induced colonic tumorigenesis through suppressed colonic mucosa cellular proliferation and increased stimulation of macrophages. J. Agric. Food Chem. 59 (24), 13337–13345. 10.1021/jf203444d 22049926

[B56] Fox J. G. Wang T. C. (2007). Inflammation, atrophy, and gastric cancer. J. Clin. Invest 117 (1), 60–69. 10.1172/JCI30111 17200707 PMC1716216

[B57] Francescone R. Hou V. Grivennikov S. I. (2014). Microbiome, inflammation, and cancer. Cancer J. 20 (3), 181–189. 10.1097/PPO.0000000000000048 24855005 PMC4112188

[B58] Gamallat Y. Meyiah A. Kuugbee E. D. Hago A. M. Chiwala G. Awadasseid A. (2016). Lactobacillus rhamnosus induced epithelial cell apoptosis, ameliorates inflammation and prevents colon cancer development in an animal model. Biomed. Pharmacother. 83, 536–541. 10.1016/j.biopha.2016.07.001 27447122

[B59] Ganal S. C. Sanos S. L. Kallfass C. Oberle K. Johner C. Kirschning C. (2012). Priming of natural killer cells by nonmucosal mononuclear phagocytes requires instructive signals from commensal microbiota. Immunity 37 (1), 171–186. 10.1016/j.immuni.2012.05.020 22749822

[B60] Garrett W. S. (2015). Cancer and the microbiota. Science 348 (6230), 80–86. 10.1126/science.aaa4972 25838377 PMC5535753

[B61] Gerner E. W. Meyskens F. L. (2004). Polyamines and cancer: old molecules, new understanding. Nat. Rev. Cancer 4 (10), 781–792. 10.1038/nrc1454 15510159

[B62] Gopalakrishnan V. Spencer C. N. Nezi L. Reuben A. Andrews M. C. Karpinets T. V. (2018). Gut microbiome modulates response to anti-PD-1 immunotherapy in melanoma patients. Science 359 (6371), 97–103. 10.1126/science.aan4236 29097493 PMC5827966

[B63] Gorski A. Weber-Dabrowska B. (2005). The potential role of endogenous bacteriophages in controlling invading pathogens. Cell Mol. Life Sci. 62 (5), 511–519. 10.1007/s00018-004-4403-6 15747058 PMC11365883

[B64] Gou H. Zeng R. Lau H. C. H. Yu J. (2024). Gut microbial metabolites: shaping future diagnosis and treatment against gastrointestinal cancer. Pharmacol. Res. 208, 107373. 10.1016/j.phrs.2024.107373 39197712

[B65] Grivennikov S. I. Wang K. Mucida D. Stewart C. A. Schnabl B. Jauch D. (2012). Adenoma-linked barrier defects and microbial products drive IL-23/IL-17-mediated tumour growth. Nature 491 (7423), 254–258. 10.1038/nature11465 23034650 PMC3601659

[B66] Groschwitz K. R. Hogan S. P. (2009). Intestinal barrier function: molecular regulation and disease pathogenesis. J. Allergy Clin. Immunol. 124 (1), 3–20. 10.1016/j.jaci.2009.05.038 19560575 PMC4266989

[B67] Guan H. Zhang X. Kuang M. Yu J. (2022). The gut-liver axis in immune remodeling of hepatic cirrhosis. Front. Immunol. 13, 946628. 10.3389/fimmu.2022.946628 37408838 PMC10319400

[B68] Guiomar de Almeida Brasiel P. Cristina Potente Dutra Luquetti S. Dutra Medeiros J. Otavio do Amaral Correa J. Barbosa Ferreira Machado A. Paula B. M. A. (2022). Kefir modulates gut microbiota and reduces DMH-associated colorectal cancer *via* regulation of intestinal inflammation in adulthood offsprings programmed by neonatal overfeeding. Food Res. Int. 152, 110708. 10.1016/j.foodres.2021.110708 35181109

[B69] Guo C. Guo D. Fang L. Sang T. Wu J. Guo C. (2021). Ganoderma lucidum polysaccharide modulates gut microbiota and immune cell function to inhibit inflammation and tumorigenesis in colon. Carbohydr. Polym. 267, 118231. 10.1016/j.carbpol.2021.118231 34119183

[B70] Hakansson A. Branning C. Molin G. Adawi D. Hagslatt M. L. Jeppsson B. (2012). Blueberry husks and probiotics attenuate colorectal inflammation and oncogenesis, and liver injuries in rats exposed to cycling DSS-treatment. PLoS One 7 (3), e33510. 10.1371/journal.pone.0033510 22457771 PMC3311639

[B71] Hanahan D. Weinberg R. A. (2011). Hallmarks of cancer: the next generation. Cell 144 (5), 646–674. 10.1016/j.cell.2011.02.013 21376230

[B72] Hannani D. Ma Y. Yamazaki T. Dechanet-Merville J. Kroemer G. Zitvogel L. (2012). Harnessing gammadelta T cells in anticancer immunotherapy. Trends Immunol. 33 (5), 199–206. 10.1016/j.it.2012.01.006 22364810

[B73] Hanus M. Parada-Venegas D. Landskron G. Wielandt A. M. Hurtado C. Alvarez K. (2021). Immune system, microbiota, and microbial metabolites: the unresolved triad in colorectal cancer microenvironment. Front. Immunol. 12, 612826. 10.3389/fimmu.2021.612826 33841394 PMC8033001

[B74] Hasko G. Kuhel D. G. Marton A. Nemeth Z. H. Deitch E. A. Szabo C. (2000). Spermine differentially regulates the production of interleukin-12 p40 and interleukin-10 and suppresses the release of the T helper 1 cytokine interferon-gamma. Shock 14 (2), 144–149. 10.1097/00024382-200014020-00012 10947158

[B75] He R. Qi P. Shu L. Ding Y. Zeng P. Wen G. (2025). Dysbiosis and extraintestinal cancers. J. Exp. Clin. Cancer Res. 44 (1), 44. 10.1186/s13046-025-03313-x 39915884 PMC11804008

[B76] Herlo L. F. Salcudean A. Sirli R. Iurciuc S. Herlo A. Nelson-Twakor A. (2024). Gut microbiota signatures in colorectal cancer as a potential diagnostic biomarker in the future: a systematic review. Int. J. Mol. Sci. 25 (14), 7937. 10.3390/ijms25147937 39063179 PMC11276678

[B77] Hill D. A. Artis D. (2010). Intestinal bacteria and the regulation of immune cell homeostasis. Annu. Rev. Immunol. 28, 623–667. 10.1146/annurev-immunol-030409-101330 20192812 PMC5610356

[B78] Holscher H. D. Caporaso J. G. Hooda S. Brulc J. M. Fahey G. C. Swanson K. S. (2015). Fiber supplementation influences phylogenetic structure and functional capacity of the human intestinal microbiome: follow-up of a randomized controlled trial. Am. J. Clin. Nutr. 101 (1), 55–64. 10.3945/ajcn.114.092064 25527750

[B79] Hu J. Wang C. Ye L. Yang W. Huang H. Meng F. (2015). Anti-tumour immune effect of oral administration of Lactobacillus plantarum to CT26 tumour-bearing mice. J. Biosci. 40 (2), 269–279. 10.1007/s12038-015-9518-4 25963256

[B80] Ibragimova S. Ramachandran R. Ali F. R. Lipovich L. Ho S. B. (2021). Dietary patterns and associated microbiome changes that promote oncogenesis. Front. Cell Dev. Biol. 9, 725821. 10.3389/fcell.2021.725821 34869313 PMC8633417

[B81] Irecta-Najera C. A. Del Rosario Huizar-Lopez M. Casas-Solis J. Castro-Felix P. Santerre A. (2017). Protective effect of Lactobacillus casei on DMH-induced Colon carcinogenesis in mice. Probiotics Antimicrob. Proteins 9 (2), 163–171. 10.1007/s12602-017-9253-2 28316010

[B82] Ivanov I. I. Atarashi K. Manel N. Brodie E. L. Shima T. Karaoz U. (2009). Induction of intestinal Th17 cells by segmented filamentous bacteria. Cell 139 (3), 485–498. 10.1016/j.cell.2009.09.033 19836068 PMC2796826

[B83] Ivleva E. A. Grivennikov S. I. (2022). Microbiota-driven mechanisms at different stages of cancer development. Neoplasia 32, 100829. 10.1016/j.neo.2022.100829 35933824 PMC9364013

[B84] Jacouton E. Chain F. Sokol H. Langella P. Bermudez-Humaran L. G. (2017). Probiotic strain Lactobacillus casei BL23 prevents colitis-associated colorectal cancer. Front. Immunol. 8, 1553. 10.3389/fimmu.2017.01553 29209314 PMC5702231

[B85] Jans M. Vereecke L. (2025). Physiological drivers of pks+ *E. coli* in colorectal cancer. Trends Microbiol. 33 (9), 1003–1017. 10.1016/j.tim.2025.04.010 40335416

[B86] Jenkins S. V. Robeson M. S. Griffin R. J. Quick C. M. Siegel E. R. Cannon M. J. (2019). Gastrointestinal tract dysbiosis enhances distal tumor progression through suppression of leukocyte trafficking. Cancer Res. 79 (23), 5999–6009. 10.1158/0008-5472.CAN-18-4108 31591154 PMC6891208

[B87] Ji X. Hou C. Zhang X. Han L. Yin S. Peng Q. (2019). Microbiome-metabolomic analysis of the impact of Zizyphus jujuba cv. muzao polysaccharides consumption on colorectal cancer mice fecal microbiota and metabolites. Int. J. Biol. Macromol. 131, 1067–1076. 10.1016/j.ijbiomac.2019.03.175 30926487

[B88] Jia W. Xie G. Jia W. (2018). Bile acid-microbiota crosstalk in gastrointestinal inflammation and carcinogenesis. Nat. Rev. Gastroenterol. Hepatol. 15 (2), 111–128. 10.1038/nrgastro.2017.119 29018272 PMC5899973

[B89] Jin W. B. Xiao L. Jeong M. Han S. J. Zhang W. Yano H. (2025). Microbiota-derived bile acids antagonize the host androgen receptor and drive anti-tumor immunity. Cell 188 (9), 2336–53 e38. 10.1016/j.cell.2025.02.029 40239649 PMC12439225

[B90] Jing Y. Y. Han Z. P. Sun K. Zhang S. S. Hou J. Liu Y. (2012). Toll-like receptor 4 signaling promotes epithelial-mesenchymal transition in human hepatocellular carcinoma induced by lipopolysaccharide. BMC Med. 10, 98. 10.1186/1741-7015-10-98 22938142 PMC3482562

[B91] Johansson M. E. Gustafsson J. K. Holmen-Larsson J. Jabbar K. S. Xia L. Xu H. (2014). Bacteria penetrate the normally impenetrable inner colon mucus layer in both murine colitis models and patients with ulcerative colitis. Gut 63 (2), 281–291. 10.1136/gutjnl-2012-303207 23426893 PMC3740207

[B92] Kang X. Liu C. Ding Y. Ni Y. Ji F. Lau H. C. H. (2023). Roseburia intestinalis generated butyrate boosts anti-PD-1 efficacy in colorectal cancer by activating cytotoxic CD8(+) T cells. Gut 72 (11), 2112–2122. 10.1136/gutjnl-2023-330291 37491158 PMC10579466

[B93] Kawai T. Akira S. (2006). Innate immune recognition of viral infection. Nat. Immunol. 7 (2), 131–137. 10.1038/ni1303 16424890

[B94] Kawamata Y. Fujii R. Hosoya M. Harada M. Yoshida H. Miwa M. (2003). A G protein-coupled receptor responsive to bile acids. J. Biol. Chem. 278 (11), 9435–9440. 10.1074/jbc.M209706200 12524422

[B95] Kazmierczak-Siedlecka K. Daca A. Fic M. van de Wetering T. Folwarski M. Makarewicz W. (2020). Therapeutic methods of gut microbiota modification in colorectal cancer management - fecal microbiota transplantation, prebiotics, probiotics, and synbiotics. Gut Microbes 11 (6), 1518–1530. 10.1080/19490976.2020.1764309 32453670 PMC7524363

[B96] Kim S. C. Tonkonogy S. L. Albright C. A. Tsang J. Balish E. J. Braun J. (2005). Variable phenotypes of enterocolitis in interleukin 10-deficient mice monoassociated with two different commensal bacteria. Gastroenterology 128 (4), 891–906. 10.1053/j.gastro.2005.02.009 15825073

[B97] Kong Y. Cao W. Xi X. Ma C. Cui L. He W. (2009). The NKG2D ligand ULBP4 binds to TCRgamma9/delta2 and induces cytotoxicity to tumor cells through both TCRgammadelta and NKG2D. Blood 114 (2), 310–317. 10.1182/blood-2008-12-196287 19436053

[B98] Koppel N. Maini Rekdal V. Balskus E. P. (2017). Chemical transformation of xenobiotics by the human gut microbiota. Science 356 (6344). 10.1126/science.aag2770 28642381 PMC5534341

[B99] Kumar A. Singh N. K. Sinha P. R. (2010). Inhibition of 1,2-dimethylhydrazine induced colon genotoxicity in rats by the administration of probiotic curd. Mol. Biol. Rep. 37 (3), 1373–1376. 10.1007/s11033-009-9519-1 19330535

[B100] Kuugbee E. D. Shang X. Gamallat Y. Bamba D. Awadasseid A. Suliman M. A. (2016). Structural change in microbiota by a probiotic cocktail enhances the gut barrier and reduces cancer *via* TLR2 signaling in a rat model of Colon cancer. Dig. Dis. Sci. 61 (10), 2908–2920. 10.1007/s10620-016-4238-7 27384052

[B101] Kvakova M. Kamlarova A. Stofilova J. Benetinova V. Bertkova I. (2022). Probiotics and postbiotics in colorectal cancer: prevention and complementary therapy. World J. Gastroenterol. 28 (27), 3370–3382. 10.3748/wjg.v28.i27.3370 36158273 PMC9346452

[B102] Le Leu R. K. Brown I. L. Hu Y. Bird A. R. Jackson M. Esterman A. (2005). A synbiotic combination of resistant starch and Bifidobacterium lactis facilitates apoptotic deletion of carcinogen-damaged cells in rat colon. J. Nutr. 135 (5), 996–1001. 10.1093/jn/135.5.996 15867271

[B103] Le Leu R. K. Hu Y. Brown I. L. Woodman R. J. Young G. P. (2010). Synbiotic intervention of Bifidobacterium lactis and resistant starch protects against colorectal cancer development in rats. Carcinogenesis 31 (2), 246–251. 10.1093/carcin/bgp197 19696163

[B104] Lee N. K. Park J. S. Park E. Paik H. D. (2007). Adherence and anticarcinogenic effects of bacillus polyfermenticus SCD in the large intestine. Lett. Appl. Microbiol. 44 (3), 274–278. 10.1111/j.1472-765X.2006.02078.x 17309504

[B105] Lee C. W. Chen H. J. Chien Y. H. Hsia S. M. Chen J. H. Shih C. K. (2019). Synbiotic combination of djulis (Chenopodium formosanum) and Lactobacillus acidophilus inhibits Colon carcinogenesis in rats. Nutrients 12 (1). 10.3390/nu12010103 31905929 PMC7019357

[B106] Lei L. Zhao L. Y. Cheng R. Zhang H. Xia M. Chen X. L. (2024). Distinct oral-associated gastric microbiota and *Helicobacter pylori* communities for spatial microbial heterogeneity in gastric cancer. mSystems 9 (7), e0008924. 10.1128/msystems.00089-24 38940519 PMC11265414

[B107] Levy M. Kolodziejczyk A. A. Thaiss C. A. Elinav E. (2017). Dysbiosis and the immune system. Nat. Rev. Immunol. 17 (4), 219–232. 10.1038/nri.2017.7 28260787

[B108] Li M. Wang B. Sun X. Tang Y. Wei X. Ge B. (2017). Upregulation of intestinal barrier function in mice with DSS-induced colitis by a defined bacterial consortium is associated with expansion of IL-17A producing gamma Delta T cells. Front. Immunol. 8, 824. 10.3389/fimmu.2017.00824 28747917 PMC5506203

[B109] Li M. van Esch B. Henricks P. A. J. Folkerts G. Garssen J. (2018). The anti-inflammatory effects of short chain fatty acids on Lipopolysaccharide- or tumor necrosis factor alpha-stimulated endothelial cells *via* activation of GPR41/43 and inhibition of HDACs. Front. Pharmacol. 9, 533. 10.3389/fphar.2018.00533 29875665 PMC5974203

[B110] Li Y. Wang S. Sun Y. Xu W. Zheng H. Wang Y. (2020). Apple polysaccharide protects ICR mice against colitis associated colorectal cancer through the regulation of microbial dysbiosis. Carbohydr. Polym. 230, 115726. 10.1016/j.carbpol.2019.115726 31887919

[B111] Li Q. Wu W. Gong D. Shang R. Wang J. Yu H. (2021a). Propionibacterium acnes overabundance in gastric cancer promote M2 polarization of macrophages *via* a TLR4/PI3K/Akt signaling. Gastric Cancer 24 (6), 1242–1253. 10.1007/s10120-021-01202-8 34076786

[B112] Li Q. Hu W. Liu W. X. Zhao L. Y. Huang D. Liu X. D. (2021b). Streptococcus thermophilus inhibits colorectal tumorigenesis through secreting beta-galactosidase. Gastroenterology 160 (4), 1179–93 e14. 10.1053/j.gastro.2020.09.003 32920015

[B113] Li Z. Xiong W. Liang Z. Wang J. Zeng Z. Kolat D. (2024). Critical role of the gut microbiota in immune responses and cancer immunotherapy. J. Hematol. Oncol. 17 (1), 33. 10.1186/s13045-024-01541-w 38745196 PMC11094969

[B114] Liang J. Li H. Chen J. He L. Du X. Zhou L. (2019). Dendrobium officinale polysaccharides alleviate colon tumorigenesis *via* restoring intestinal barrier function and enhancing anti-tumor immune response. Pharmacol. Res. 148, 104417. 10.1016/j.phrs.2019.104417 31473343

[B115] Liboredo J. C. Anastacio L. R. Peluzio Mdo C. Valente F. X. Penido L. C. Nicoli J. R. (2013). Effect of probiotics on the development of dimethylhydrazine-induced preneoplastic lesions in the mice colon. Acta Cir. Bras. 28 (5), 367–372. 10.1590/s0102-86502013000500008 23702939

[B116] Liu D. Zhang R. Chen S. Sun B. Zhang K. (2022). Analysis of gastric microbiome reveals three distinctive microbial communities associated with the occurrence of gastric cancer. BMC Microbiol. 22 (1), 184. 10.1186/s12866-022-02594-y 35870901 PMC9308235

[B117] Lopez-Siles M. Duncan S. H. Garcia-Gil L. J. Martinez-Medina M. (2017). Faecalibacterium prausnitzii: from microbiology to diagnostics and prognostics. ISME J. 11 (4), 841–852. 10.1038/ismej.2016.176 28045459 PMC5364359

[B118] Louis P. Hold G. L. Flint H. J. (2014). The gut microbiota, bacterial metabolites and colorectal cancer. Nat. Rev. Microbiol. 12 (10), 661–672. 10.1038/nrmicro3344 25198138

[B119] Ma C. Han M. Heinrich B. Fu Q. Zhang Q. Sandhu M. (2018). Gut microbiome-mediated bile acid metabolism regulates liver cancer *via* NKT cells. Science 360 (6391). 10.1126/science.aan5931 29798856 PMC6407885

[B120] Ma H. Yang L. Liang Y. Liu F. Hu J. Zhang R. (2024). B. Thetaiotaomicron-derived acetic acid modulate immune microenvironment and tumor growth in hepatocellular carcinoma. Gut Microbes 16 (1), 2297846. 10.1080/19490976.2023.2297846 38270111 PMC10813637

[B121] Malik A. Sharma D. Zhu Q. Karki R. Guy C. S. Vogel P. (2016). IL-33 regulates the IgA-microbiota axis to restrain IL-1alpha-dependent colitis and tumorigenesis. J. Clin. Invest 126 (12), 4469–4481. 10.1172/JCI88625 27775548 PMC5127671

[B122] Maran R. R. Thomas A. Roth M. Sheng Z. Esterly N. Pinson D. (2009). Farnesoid X receptor deficiency in mice leads to increased intestinal epithelial cell proliferation and tumor development. J. Pharmacol. Exp. Ther. 328 (2), 469–477. 10.1124/jpet.108.145409 18981289 PMC2682273

[B123] Marzo F. Jauregui P. Barrenetxe J. Martinez-Penuela A. Ibanez F. C. Milagro F. I. (2022). Effect of a diet supplemented with sphingomyelin and probiotics on Colon cancer development in mice. Probiotics Antimicrob. Proteins 14 (3), 407–414. 10.1007/s12602-022-09916-6 35112298 PMC9076719

[B124] Matson V. Fessler J. Bao R. Chongsuwat T. Zha Y. Alegre M. L. (2018). The commensal microbiome is associated with anti-PD-1 efficacy in metastatic melanoma patients. Science 359 (6371), 104–108. 10.1126/science.aao3290 29302014 PMC6707353

[B125] Miao W. Wu X. Wang K. Wang W. Wang Y. Li Z. (2016). Sodium butyrate promotes reassembly of tight junctions in Caco-2 monolayers involving inhibition of MLCK/MLC2 pathway and phosphorylation of PKCbeta2. Int. J. Mol. Sci. 17 (10). 10.3390/ijms17101696 27735862 PMC5085728

[B126] Moschen A. R. Gerner R. R. Wang J. Klepsch V. Adolph T. E. Reider S. J. (2016). Lipocalin 2 protects from inflammation and tumorigenesis associated with gut microbiota alterations. Cell Host Microbe 19 (4), 455–469. 10.1016/j.chom.2016.03.007 27078067

[B127] Mousavi Jam S. A. Morshedi M. Yari Khosroushahi A. Eftekharsadat A. T. Alipour M. Alipour B. (2020). Preventive and tumor-suppressive effects of Lactobacillus paracasei X12 in rat model of colorectal cancer. Iran. J. Pharm. Res. 19 (4), 330–342. 10.22037/ijpr.2019.112135.13547 33841546 PMC8019866

[B128] Murray Stewart T. Dunston T. T. Woster P. M. Casero R. A. Jr (2018). Polyamine catabolism and oxidative damage. J. Biol. Chem. 293 (48), 18736–18745. 10.1074/jbc.TM118.003337 30333229 PMC6290137

[B129] Narushima S. Sakata T. Hioki K. Itoh T. Nomura T. Itoh K. (2010). Inhibitory effect of yogurt on aberrant crypt foci formation in the rat colon and colorectal tumorigenesis in RasH2 mice. Exp. Anim. 59 (4), 487–494. 10.1538/expanim.59.487 20660995

[B130] Neurath M. F. Artis D. Becker C. (2025). The intestinal barrier: a pivotal role in health, inflammation, and cancer. Lancet Gastroenterol. Hepatol. 10 (6), 573–592. 10.1016/S2468-1253(24)00390-X 40086468

[B131] Nobels A. van Marcke C. Jordan B. F. Van Hul M. Cani P. D. (2025). The gut microbiome and cancer: from tumorigenesis to therapy. Nat. Metab. 7 (5), 895–917. 10.1038/s42255-025-01287-w 40329009

[B132] O'Mahony C. Scully P. O'Mahony D. Murphy S. O'Brien F. Lyons A. (2008). Commensal-induced regulatory T cells mediate protection against pathogen-stimulated NF-kappaB activation. PLoS Pathog. 4 (8), e1000112. 10.1371/journal.ppat.1000112 18670628 PMC2474968

[B133] Pan M. Wan C. Xie Q. Huang R. Tao X. Shah N. P. (2016). Changes in gastric microbiota induced by *Helicobacter pylori* infection and preventive effects of Lactobacillus plantarum ZDY 2013 against such infection. J. Dairy Sci. 99 (2), 970–981. 10.3168/jds.2015-10510 26709179

[B134] Park E. Jeon G. I. Park J. S. Paik H. D. (2007). A probiotic strain of bacillus polyfermenticus reduces DMH induced precancerous lesions in F344 male rat. Biol. Pharm. Bull. 30 (3), 569–574. 10.1248/bpb.30.569 17329858

[B135] Payne C. M. Bernstein C. Dvorak K. Bernstein H. (2008). Hydrophobic bile acids, genomic instability, Darwinian selection, and colon carcinogenesis. Clin. Exp. Gastroenterol. 1, 19–47. 10.2147/ceg.s4343 21677822 PMC3108627

[B136] Pegg A. E. (2013). Toxicity of polyamines and their metabolic products. Chem. Res. Toxicol. 26 (12), 1782–1800. 10.1021/tx400316s 24224555

[B137] Perez M. Buey B. Corral P. Giraldos D. Latorre E. (2024). Microbiota-derived short-chain fatty acids boost antitumoral natural killer cell activity. J. Clin. Med. 13 (13). 10.3390/jcm13133885 38999461 PMC11242436

[B138] Peuker K. Muff S. Wang J. Kunzel S. Bosse E. Zeissig Y. (2016). Epithelial calcineurin controls microbiota-dependent intestinal tumor development. Nat. Med. 22 (5), 506–515. 10.1038/nm.4072 27043494 PMC5570457

[B139] Pithva S. P. Ambalam P. S. Ramoliya J. M. Dave J. M. Vyas B. R. (2015). Antigenotoxic and antimutagenic activities of probiotic Lactobacillus rhamnosus Vc against N-Methyl-N'-Nitro-N-Nitrosoguanidine. Nutr. Cancer 67 (7), 1142–1150. 10.1080/01635581.2015.1073751 26312410

[B140] Plaza-Diaz J. Alvarez-Mercado A. I. Ruiz-Marin C. M. Reina-Perez I. Perez-Alonso A. J. Sanchez-Andujar M. B. (2019). Association of breast and gut microbiota dysbiosis and the risk of breast cancer: a case-control clinical study. BMC Cancer 19 (1), 495. 10.1186/s12885-019-5660-y 31126257 PMC6534876

[B141] Plummer M. de Martel C. Vignat J. Ferlay J. Bray F. Franceschi S. (2016). Global burden of cancers attributable to infections in 2012: a synthetic analysis. Lancet Glob. Health 4 (9), e609–e616. 10.1016/S2214-109X(16)30143-7 27470177

[B142] Pool-Zobel B. van Loo J. Rowland I. Roberfroid M. B. (2002). Experimental evidences on the potential of prebiotic fructans to reduce the risk of colon cancer. Br. J. Nutr. 87 (Suppl. 2), S273–S281. 10.1079/BJNBJN/2002548 12088529

[B143] Purohit D. H. Hassan A. N. Bhatia E. Zhang X. Dwivedi C. (2009). Rheological, sensorial, and chemopreventive properties of milk fermented with exopolysaccharide-producing lactic cultures. J. Dairy Sci. 92 (3), 847–856. 10.3168/jds.2008-1256 19233777

[B144] Rafii F. Cerniglia C. E. (1995). Reduction of azo dyes and nitroaromatic compounds by bacterial enzymes from the human intestinal tract. Environ. Health Perspect. 103 (Suppl. 5), 17–19. 10.1289/ehp.95103s417 8565901 PMC1519296

[B145] Ramos-Molina B. Queipo-Ortuno M. I. Lambertos A. Tinahones F. J. Penafiel R. (2019). Dietary and gut microbiota polyamines in Obesity- and age-related diseases. Front. Nutr. 6, 24. 10.3389/fnut.2019.00024 30923709 PMC6426781

[B146] Reis S. K. Socca E. A. R. de Souza B. R. Genaro S. C. Duran N. Favaro W. J. (2024). Effects of probiotic supplementation on chronic inflammatory process modulation in colorectal carcinogenesis. Tissue Cell 87, 102293. 10.1016/j.tice.2023.102293 38244400

[B147] Roje B. Zhang B. Mastrorilli E. Kovacic A. Susak L. Ljubenkov I. (2024). Gut microbiota carcinogen metabolism causes distal tissue tumours. Nature 632 (8027), 1137–1144. 10.1038/s41586-024-07754-w 39085612 PMC11358042

[B148] Roy U. de Oliveira R. S. Galvez E. J. C. Gronow A. Basic M. Perez L. G. (2021). Induction of IL-22-Producing CD4+ T cells by segmented filamentous bacteria independent of classical Th17 cells. Front. Immunol. 12, 671331. 10.3389/fimmu.2021.671331 34566952 PMC8456099

[B149] Schiller J. T. Lowy D. R. (2021). An introduction to virus infections and human cancer. Recent Results Cancer Res. 217, 1–11. 10.1007/978-3-030-57362-1_1 33200359 PMC8336782

[B150] Schneider K. M. Mohs A. Gui W. Galvez E. J. C. Candels L. S. Hoenicke L. (2022). Imbalanced gut microbiota fuels hepatocellular carcinoma development by shaping the hepatic inflammatory microenvironment. Nat. Commun. 13 (1), 3964. 10.1038/s41467-022-31312-5 35803930 PMC9270328

[B151] Schwabe R. F. Jobin C. (2013). The microbiome and cancer. Nat. Rev. Cancer 13 (11), 800–812. 10.1038/nrc3610 24132111 PMC3986062

[B152] Scott A. J. Alexander J. L. Merrifield C. A. Cunningham D. Jobin C. Brown R. (2019). International cancer microbiome consortium consensus statement on the role of the human microbiome in carcinogenesis. Gut 68 (9), 1624–1632. 10.1136/gutjnl-2019-318556 31092590 PMC6709773

[B153] Sears C. L. Pardoll D. M. (2011). Perspective: alpha-bugs, their microbial partners, and the link to colon cancer. J. Infect. Dis. 203 (3), 306–311. 10.1093/jinfdis/jiq061 21208921 PMC3071114

[B154] Sender R. Fuchs S. Milo R. (2016). Revised estimates for the number of human and bacteria cells in the body. PLoS Biol. 14 (8), e1002533. 10.1371/journal.pbio.1002533 27541692 PMC4991899

[B155] Sharaf L. K. Sharma M. Chandel D. Shukla G. (2018). Prophylactic intervention of probiotics (L.acidophilus, L.rhamnosus GG) and celecoxib modulate Bax-mediated apoptosis in 1,2-dimethylhydrazine-induced experimental colon carcinogenesis. BMC Cancer 18 (1), 1111. 10.1186/s12885-018-4999-9 30424722 PMC6234654

[B156] Singh N. Gurav A. Sivaprakasam S. Brady E. Padia R. Shi H. (2014). Activation of Gpr109a, receptor for niacin and the commensal metabolite butyrate, suppresses colonic inflammation and carcinogenesis. Immunity 40 (1), 128–139. 10.1016/j.immuni.2013.12.007 24412617 PMC4305274

[B157] Singh V. Yeoh B. S. Chassaing B. Zhang B. Saha P. Xiao X. (2016). Microbiota-inducible innate immune, siderophore binding protein lipocalin 2 is critical for intestinal homeostasis. Cell Mol. Gastroenterol. Hepatol. 2 (4), 482–98 e6. 10.1016/j.jcmgh.2016.03.007 27458605 PMC4957954

[B158] Smith P. M. Howitt M. R. Panikov N. Michaud M. Gallini C. A. Bohlooly Y. M. (2013). The microbial metabolites, short-chain fatty acids, regulate colonic treg cell homeostasis. Science 341 (6145), 569–573. 10.1126/science.1241165 23828891 PMC3807819

[B159] So D. Whelan K. Rossi M. Morrison M. Holtmann G. Kelly J. T. (2018). Dietary fiber intervention on gut microbiota composition in healthy adults: a systematic review and meta-analysis. Am. J. Clin. Nutr. 107 (6), 965–983. 10.1093/ajcn/nqy041 29757343

[B160] Song M. Chan A. T. (2019). Environmental factors, gut microbiota, and colorectal cancer prevention. Clin. Gastroenterol. Hepatol. 17 (2), 275–289. 10.1016/j.cgh.2018.07.012 30031175 PMC6314893

[B161] Song M. Chan A. T. Fuchs C. S. Ogino S. Hu F. B. Mozaffarian D. (2014). Dietary intake of fish, omega-3 and omega-6 fatty acids and risk of colorectal cancer: a prospective study in U.S. men and women. Int. J. Cancer 135 (10), 2413–2423. 10.1002/ijc.28878 24706410 PMC4159425

[B162] Song M. Zhang X. Meyerhardt J. A. Giovannucci E. L. Ogino S. Fuchs C. S. (2017). Marine omega-3 polyunsaturated fatty acid intake and survival after colorectal cancer diagnosis. Gut 66 (10), 1790–1796. 10.1136/gutjnl-2016-311990 27436272 PMC5247396

[B163] Su A. C. Y. Ding X. Lau H. C. H. Kang X. Li Q. Wang X. (2024). Lactococcus lactis HkyuLL 10 suppresses colorectal tumourigenesis and restores gut microbiota through its generated alpha-mannosidase. Gut 73 (9), 1478–1488. 10.1136/gutjnl-2023-330835 38599786 PMC11347254

[B164] Sui Y. Wu J. Chen J. (2021). The role of gut microbial beta-Glucuronidase in estrogen reactivation and breast cancer. Front. Cell Dev. Biol. 9, 631552. 10.3389/fcell.2021.631552 34458248 PMC8388929

[B165] Sun H. Zhai Q. Liu J. Shi K. Fan W. (2025). Interplay between the gut microbiota, its metabolites and carcinogens. Clin. Transl. Oncol. 27 (11), 4103–4116. 10.1007/s12094-025-03920-2 40358880

[B166] Takiishi T. Fenero C. I. M. Camara N. O. S. (2017). Intestinal barrier and gut microbiota: shaping our immune responses throughout life. Tissue Barriers 5 (4), e1373208. 10.1080/21688370.2017.1373208 28956703 PMC5788425

[B167] Talero E. Bolivar S. Avila-Roman J. Alcaide A. Fiorucci S. Motilva V. (2015). Inhibition of chronic ulcerative colitis-associated adenocarcinoma development in mice by VSL#3. Inflamm. Bowel Dis. 21 (5), 1027–1037. 10.1097/MIB.0000000000000346 25793324

[B168] Tougeron D. Fauquembergue E. Latouche J. B. (2013). Immune response and colorectal cancer. Bull. Cancer 100 (3), 283–294. 10.1684/bdc.2013.1716 23501583

[B169] Tsilimigras M. C. Fodor A. Jobin C. (2017). Carcinogenesis and therapeutics: the microbiota perspective. Nat. Microbiol. 2, 17008. 10.1038/nmicrobiol.2017.8 28225000 PMC6423540

[B170] Urbanska A. M. Bhathena J. Cherif S. Prakash S. (2016). Orally delivered microencapsulated probiotic formulation favorably impacts polyp formation in APC (Min/+) model of intestinal carcinogenesis. Artif. Cells Nanomed Biotechnol. 44 (1), 1–11. 10.3109/21691401.2014.898647 25060720

[B171] van Nood E. Vrieze A. Nieuwdorp M. Fuentes S. Zoetendal E. G. de Vos W. M. (2013). Duodenal infusion of donor feces for recurrent *Clostridium difficile* . N. Engl. J. Med. 368 (5), 407–415. 10.1056/NEJMoa1205037 23323867

[B172] Vavassori P. Mencarelli A. Renga B. Distrutti E. Fiorucci S. (2009). The bile acid receptor FXR is a modulator of intestinal innate immunity. J. Immunol. 183 (10), 6251–6261. 10.4049/jimmunol.0803978 19864602

[B173] Verma A. Shukla G. (2013). Probiotics Lactobacillus rhamnosus GG, Lactobacillus acidophilus suppresses DMH-induced procarcinogenic fecal enzymes and preneoplastic aberrant crypt foci in early colon carcinogenesis in sprague dawley rats. Nutr. Cancer 65 (1), 84–91. 10.1080/01635581.2013.741746 23368917

[B174] Vipperla K. O'Keefe S. J. (2016). Diet, microbiota, and dysbiosis: a 'recipe' for colorectal cancer. Food Funct. 7 (4), 1731–1740. 10.1039/c5fo01276g 26840037 PMC6501806

[B175] Walia S. Kamal R. Dhawan D. K. Kanwar S. S. (2018). Chemoprevention by probiotics during 1,2-Dimethylhydrazine-Induced Colon carcinogenesis in rats. Dig. Dis. Sci. 63 (4), 900–909. 10.1007/s10620-018-4949-z 29427224

[B176] Wang N. Fang J. Y. (2023). Fusobacterium nucleatum, a key pathogenic factor and microbial biomarker for colorectal cancer. Trends Microbiol. 31 (2), 159–172. 10.1016/j.tim.2022.08.010 36058786

[B177] Wang L. Cao H. Liu L. Wang B. Walker W. A. Acra S. A. (2014a). Activation of epidermal growth factor receptor mediates mucin production stimulated by p40, a Lactobacillus rhamnosus GG-derived protein. J. Biol. Chem. 289 (29), 20234–20244. 10.1074/jbc.M114.553800 24895124 PMC4106339

[B178] Wang Z. K. Yang Y. S. Chen Y. Yuan J. Sun G. Peng L. H. (2014b). Intestinal microbiota pathogenesis and fecal microbiota transplantation for inflammatory bowel disease. World J. Gastroenterol. 20 (40), 14805–14820. 10.3748/wjg.v20.i40.14805 25356041 PMC4209544

[B179] Wang T. Zheng J. Dong S. Ismael M. Shan Y. Wang X. (2022a). Lacticaseibacillus rhamnosus LS8 ameliorates azoxymethane/dextran sulfate sodium-induced colitis-associated tumorigenesis in mice *via* regulating gut microbiota and inhibiting inflammation. Probiotics Antimicrob. Proteins 14 (5), 947–959. 10.1007/s12602-022-09967-9 35788907

[B180] Wang T. Zhang L. Wang P. Liu Y. Wang G. Shan Y. (2022b). Lactobacillus coryniformis MXJ32 administration ameliorates azoxymethane/dextran sulfate sodium-induced colitis-associated colorectal cancer *via* reshaping intestinal microenvironment and alleviating inflammatory response. Eur. J. Nutr. 61 (1), 85–99. 10.1007/s00394-021-02627-8 34185157

[B181] Wensel C. R. Pluznick J. L. Salzberg S. L. Sears C. L. (2022). Next-generation sequencing: insights to advance clinical investigations of the microbiome. J. Clin. Invest 132 (7), e154944. 10.1172/JCI154944 35362479 PMC8970668

[B182] Winkler E. S. Thackray L. B. (2019). A long-distance relationship: the commensal gut microbiota and systemic viruses. Curr. Opin. Virol. 37, 44–51. 10.1016/j.coviro.2019.05.009 31226645 PMC6768733

[B183] Wong C. C. Yu J. (2023). Gut microbiota in colorectal cancer development and therapy. Nat. Rev. Clin. Oncol. 20 (7), 429–452. 10.1038/s41571-023-00766-x 37169888

[B184] Wong S. H. Zhao L. Zhang X. Nakatsu G. Han J. Xu W. (2017). Gavage of fecal samples from patients with colorectal cancer promotes intestinal carcinogenesis in germ-free and conventional mice. Gastroenterology 153 (6), 1621–33 e6. 10.1053/j.gastro.2017.08.022 28823860

[B185] Wu H. J. Wu E. (2012). The role of gut microbiota in immune homeostasis and autoimmunity. Gut Microbes 3 (1), 4–14. 10.4161/gmic.19320 22356853 PMC3337124

[B186] Wu S. Rhee K. J. Albesiano E. Rabizadeh S. Wu X. Yen H. R. (2009). A human colonic commensal promotes colon tumorigenesis *via* activation of T helper type 17 T cell responses. Nat. Med. 15 (9), 1016–1022. 10.1038/nm.2015 19701202 PMC3034219

[B187] Wu P. Wu D. Ni C. Ye J. Chen W. Hu G. (2014). gammadeltaT17 cells promote the accumulation and expansion of myeloid-derived suppressor cells in human colorectal cancer. Immunity 40 (5), 785–800. 10.1016/j.immuni.2014.03.013 24816404 PMC4716654

[B188] Wu D. Wu P. Qiu F. Wei Q. Huang J. (2017). Human gammadeltaT-cell subsets and their involvement in tumor immunity. Cell Mol. Immunol. 14 (3), 245–253. 10.1038/cmi.2016.55 27890919 PMC5360884

[B189] Wu Z. Pfeiffer R. M. Byrd D. A. Wan Y. Ansong D. Clegg-Lamptey J. N. (2023). Associations of circulating estrogens and estrogen metabolites with fecal and oral microbiome in postmenopausal women in the Ghana breast health study. Microbiol. Spectr. 11 (4), e0157223. 10.1128/spectrum.01572-23 37341612 PMC10433996

[B190] Wu Y. Shen N. Hope C. Noh H. I. Richardson B. N. Swartz M. C. (2024). A systematic review of the gut microbiome, metabolites, and multi-omics biomarkers across the colorectal cancer care continuum. Benef. Microbes 15 (6), 539–563. 10.1163/18762891-bja00026 39147373

[B191] Xie G. Wang X. Liu P. Wei R. Chen W. Rajani C. (2016). Distinctly altered gut microbiota in the progression of liver disease. Oncotarget 7 (15), 19355–19366. 10.18632/oncotarget.8466 27036035 PMC4991388

[B192] Xu H. Hiraishi K. Kurahara L. H. Nakano-Narusawa Y. Li X. Hu Y. (2021). Inhibitory effects of breast milk-derived Lactobacillus rhamnosus Probio-M9 on colitis-associated carcinogenesis by restoration of the gut microbiota in a mouse model. Nutrients 13 (4), 1143. 10.3390/nu13041143 33808480 PMC8065529

[B193] Yang B. Wang F. L. Ren X. L. Li D. (2014). Biospecimen long-chain N-3 PUFA and risk of colorectal cancer: a meta-analysis of data from 60,627 individuals. PLoS One 9 (11), e110574. 10.1371/journal.pone.0110574 25375637 PMC4222788

[B194] Yu Y. Zhong W. (2022). Interaction of microbiome and immunity in tumorigenesis and clinical treatment. Biomed. Pharmacother. 156, 113894. 10.1016/j.biopha.2022.113894 36274461

[B195] Yu J. Li S. Guo J. Xu Z. Zheng J. Sun X. (2020). Farnesoid X receptor antagonizes wnt/beta-catenin signaling in colorectal tumorigenesis. Cell Death Dis. 11 (8), 640. 10.1038/s41419-020-02819-w 32807788 PMC7431544

[B196] Yu Q. Newsome R. C. Beveridge M. Hernandez M. C. Gharaibeh R. Z. Jobin C. (2022). Intestinal microbiota modulates pancreatic carcinogenesis through intratumoral natural killer cells. Gut Microbes 14 (1), 2112881. 10.1080/19490976.2022.2112881 35980869 PMC9397420

[B197] Yu J. Li L. Tao X. Chen Y. Dong D. (2024). Metabolic interactions of host-gut microbiota: new possibilities for the precise diagnosis and therapeutic discovery of gastrointestinal cancer in the future-A review. Crit. Rev. Oncol. Hematol. 203, 104480. 10.1016/j.critrevonc.2024.104480 39154670

[B198] Zeng R. Gou H. Lau H. C. H. Yu J. (2024). Stomach microbiota in gastric cancer development and clinical implications. Gut 73 (12), 2062–2073. 10.1136/gutjnl-2024-332815 38886045 PMC11672014

[B199] Zhang M. Fan X. Fang B. Zhu C. Zhu J. Ren F. (2015). Effects of Lactobacillus salivarius ren on cancer prevention and intestinal microbiota in 1, 2-dimethylhydrazine-induced rat model. J. Microbiol. 53 (6), 398–405. 10.1007/s12275-015-5046-z 26025172

[B200] Zhang Z. Tang H. Chen P. Xie H. Tao Y. (2019). Demystifying the manipulation of host immunity, metabolism, and extraintestinal tumors by the gut microbiome. Signal Transduct. Target Ther. 4, 41. 10.1038/s41392-019-0074-5 31637019 PMC6799818

[B201] Zhang Q. Zhao Q. Li T. Lu L. Wang F. Zhang H. (2023). Lactobacillus plantarum-derived indole-3-lactic acid ameliorates colorectal tumorigenesis *via* epigenetic regulation of CD8(+) T cell immunity. Cell Metab. 35 (6), 943–60 e9. 10.1016/j.cmet.2023.04.015 37192617

[B202] Zhao L. Y. Mei J. X. Yu G. Lei L. Zhang W. H. Liu K. (2023). Role of the gut microbiota in anticancer therapy: from molecular mechanisms to clinical applications. Signal Transduct. Target Ther. 8 (1), 201. 10.1038/s41392-023-01406-7 37179402 PMC10183032

[B203] Zheng X. Zhao A. Xie G. Chi Y. Zhao L. Li H. (2013). Melamine-induced renal toxicity is mediated by the gut microbiota. Sci. Transl. Med. 5 (172), 172ra22. 10.1126/scitranslmed.3005114 23408055

[B204] Zhu J. Zhu C. Ge S. Zhang M. Jiang L. Cui J. (2014). Lactobacillus salivarius ren prevent the early colorectal carcinogenesis in 1, 2-dimethylhydrazine-induced rat model. J. Appl. Microbiol. 117 (1), 208–216. 10.1111/jam.12499 24754742

